# Common Variants in *CDKN2B-AS1* Associated with Optic-Nerve Vulnerability of Glaucoma Identified by Genome-Wide Association Studies in Japanese

**DOI:** 10.1371/journal.pone.0033389

**Published:** 2012-03-12

**Authors:** Masakazu Nakano, Yoko Ikeda, Yuichi Tokuda, Masahiro Fuwa, Natsue Omi, Morio Ueno, Kojiro Imai, Hiroko Adachi, Masaaki Kageyama, Kazuhiko Mori, Shigeru Kinoshita, Kei Tashiro

**Affiliations:** 1 Department of Genomic Medical Sciences, Kyoto Prefectural University of Medicine, Kyoto, Japan; 2 Department of Ophthalmology, Kyoto Prefectural University of Medicine, Kyoto, Japan; 3 Research and Development Center, Santen Pharmaceutical Co. Ltd., Nara, Japan; Peninsula College of Medicine and Dentistry, United Kingdom

## Abstract

**Background:**

To date, only a small portion of the genetic variation for primary open-angle glaucoma (POAG), the major type of glaucoma, has been elucidated.

**Methods and Principal Findings:**

We examined our two data sets of the genome-wide association studies (GWAS) derived from a total of 2,219 Japanese subjects. First, we performed a GWAS by analyzing 653,519 autosomal common single-nucleotide polymorphisms (SNPs) in 833 POAG patients and 686 controls. As a result, five variants that passed the Bonferroni correction were identified in *CDKN2B-AS1* on chromosome 9p21.3, which was already reported to be a significant locus in the Caucasian population. Moreover, we combined the data set with our previous GWAS data set derived from 411 POAG patients and 289 controls by the Mantel-Haenszel test, and all of the combined variants showed stronger association with POAG (*P*<5.8×10^−10^). We then subdivided the case groups into two subtypes based on the value of intraocular pressure (IOP)—POAG with high IOP (high pressure glaucoma, HPG) and that with normal IOP (normal pressure glaucoma, NPG)—and performed the GWAS using the two data sets, as the prevalence of NPG in Japanese is much higher than in Caucasians. The results suggested that the variants from the same *CDKN2B-AS1* locus were likely to be significant for NPG patients.

**Conclusions and Significance:**

In this study, we successfully identified POAG-associated variants in the *CDKN2B-AS1* locus using a Japanese population, i.e., variants originally reported as being associated with the Caucasian population. Although we cannot rule out that the significance could be due to the differences in sample size between HPG and NPG, the variants could be associated specifically with the vulnerability of the optic nerve to IOP, which is useful for investigating the etiology of glaucoma.

## Introduction

Glaucoma is a neurodegenerative ocular disease and one of the leading causes of irreversible blindness worldwide [1]. It is characterized by the progressive loss of retinal ganglion cells and optic nerve axons, resulting in visual field defects [2]. One of the well-known major risk factors for glaucoma is elevated intraocular pressure (IOP) [2]. Thus, the measurement of IOP is routinely involved in the diagnosis of glaucoma. In fact, the IOP level has been applied to subdivide the most common form of glaucoma, primary open-angle glaucoma (POAG), into two subtypes [3]: POAG with high (≥22 mmHg) IOP (POAG/HPG, high pressure glaucoma; hereafter referred to as “HPG”) and with normal (<22 mmHg) IOP (POAG/NPG, normal pressure glaucoma; hereafter referred to as “NPG”). Interestingly, ∼92% of the Japanese POAG patients are categorized into the NPG subtype [4], whereas ∼41% of Caucasian POAG patients are categorized as NPG [5], thus showing a unique epidemiological distribution of Japanese patients compared with other ethnic groups.

Aside from IOP measurements, the diagnosis of glaucoma is commonly made by observing optic nerve degeneration and visual field defects by means of fundus examinations and visual field tests, respectively. In the case of HPG, early drug treatment to lower IOP immediately following the onset of visual field damage has been shown to be quite effective in slowing the irreversible progression toward blindness [6], [7]. In contrast, since NPG patients show normal IOP, they are often misdiagnosed. Therefore, both fundus examinations of the optic nerve and visual field tests are critical for the proper diagnosis of NPG patients. However, due to the restriction of the healthcare expenditure to include those examinations into a person's regular medical checkup, especially in the preclinical state of glaucoma, it would be of great benefit if the risk of developing glaucoma could be ascertained based on a simple blood test to assess the genetic markers for the disease.

Since glaucoma shows familial aggregation and its prevalence varies between individuals of different ethnicities, it has been theorized that genetic factors play a significant role in the pathogenesis of glaucoma [8], [9]. Therefore, several institutions are making profound efforts to discover single-nucleotide variants for glaucoma by conducting a genome-wide association study (GWAS) [10]–[14], and there are a few published reports of the association of particular loci, such as the *CAV1*/*CAV2* locus (7q31.1) [13] and the *TMCO1* or *CDKN2B-AS1* loci (1q24.1 or 9p21.3, respectively) [14], with POAG using Caucasian subjects. However, it appears that a controversy still exists as to determining the authentic variants associating with POAG, even within the same ethnicity of European descent [14], [15]. We previously reported a GWAS and the subsequent follow-up study focused on the high-ranked variants identified in the initial population using in total of 1,575 Japanese subjects [10]. We identified six variants located in three loci, 1q43, 10p12.31, and 12q21.31, on the chromosome which were modestly associated with POAG, although the association results were not reproducible with a different population of different ethnicities [13], [16], [17]. In that previous study, we combined the patients from both HPG and NPG subtypes as a single case group in order to increase the statistical power [10]. Therefore, the genetic loci that were identified are most likely to be components of the molecular mechanism shared by both subtypes. Another Japanese group performed a GWAS [12] and discovered variants in *SRBD1* on 2p21 and *ELOVL5* on 6p12.1 that were associated with NPG (in that study, NPG was referred to as “normal tension glaucoma”) [18]. However, since the findings in that study were derived from a single population, replication studies to support those findings still need to be conducted.

In this present study, we examined two independent GWAS data sets, and then performed a meta-analysis by combining the data sets in order to discover authentic genetic markers for POAG, HPG, or NPG. We succeeded in identifying some significant variants in the *CDKN2B-AS1* locus using a Japanese population, as that locus has also been shown to be significantly associated with POAG in Caucasians [14]. Moreover, the variants that passed the Bonferroni correction seemed to be associated with POAG and POAG/NPG, thus suggesting that the locus potentially affects the NPG, rather than the HPG, subtype of POAG. Although we still need to confirm our findings by use of a larger HPG cohort in order to increase the statistical power, the variants identified in this study could be associated specifically with the vulnerability of the optic nerve to IOP, thus enabling us to investigate the etiology of glaucoma.

## Results

**Figure 1:**
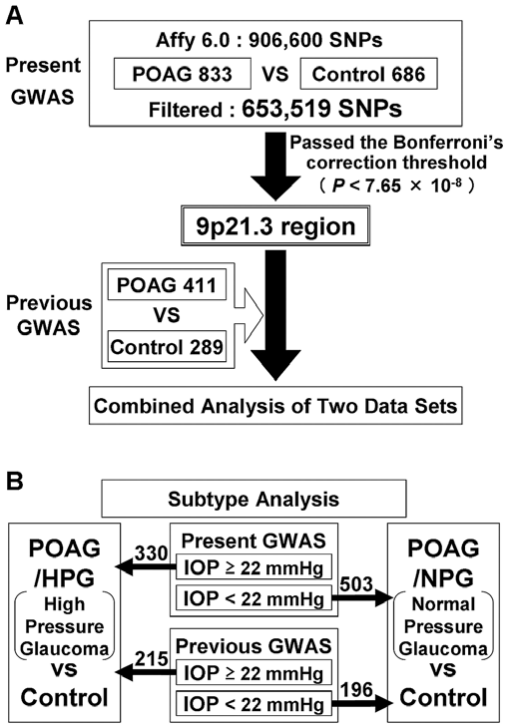
Study design. (A) We first performed the “Present GWAS” for POAG and identified the 9p21.3 locus. This result was confirmed by the combined analysis with the “Previous GWAS”. (B) We then subdivided the POAG subjects into two subtypes, POAG/HPG and POAG/NPG, and each group was analyzed by combining the two data sets in order to investigate the differences of statistical significance in the 9p21.3 locus.

**Figure 2:**
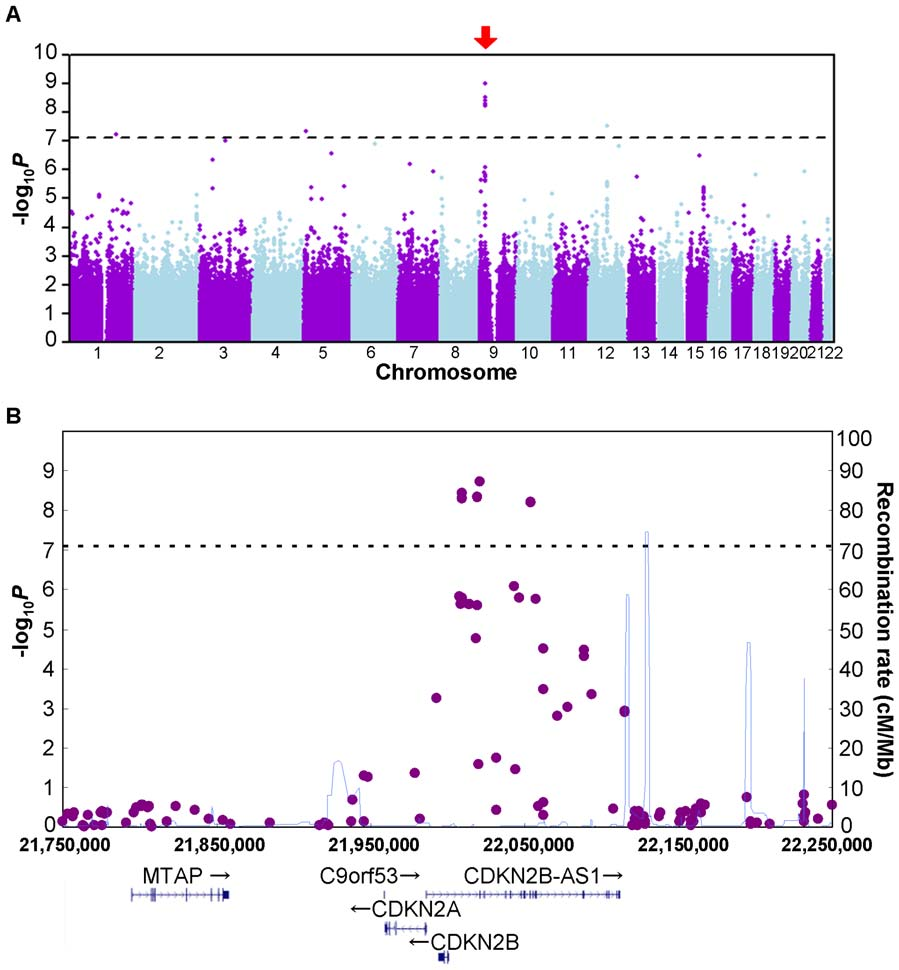
Association results of the Present GWAS. (A) The SNPs with a strong association appeared to exist in a cluster on chromosome 9 (red arrow). (B) The five significant SNPs that passed the Bonferroni correction threshold (horizontal dotted line) were identified in *CDKN2B-AS1* on the 9p21 locus. SNP positions followed the NCBI Build 36 coordinates. The genetic recombination rates (cM/Mb) estimated by HapMap Project [31] in Release 22 are indicated by the thin blue line.

We first performed a GWAS in 833 POAG patients and 686 control subjects (hereafter referred to as “Present GWAS”; Figure 1A) who were selected and divided into case and control groups based on our strict diagnosis criteria. After genotyping, 653,519 SNPs passed the quality controls and were used for the association study (Figure 1A). In a quantile-quantile (Q-Q) plot (Figure S1A), the genomic inflation factor (*λ*) showed 1.021, suggesting that the population substructure should not have any substantial effects on the association analysis. Under these conditions, we obtained 8 genome-wide significant SNPs (Table S1) that passed the Bonferroni correction threshold (0.05/653,519 = 7.65×10^−8^), of which 5 SNPs were located in *CDKN2B-AS1* on chromosome 9p21.3 (Figure 2). A few SNPs that showed modest to strong association were distributed across a single linkage disequilibrium (LD) block located in the locus (Figure S2). The remaining genome-wide significant SNPs identified from different chromosomes turned out to be genotyping errors due to the poor 2D clusters (Figure S3B–D).

**Figure 3:**
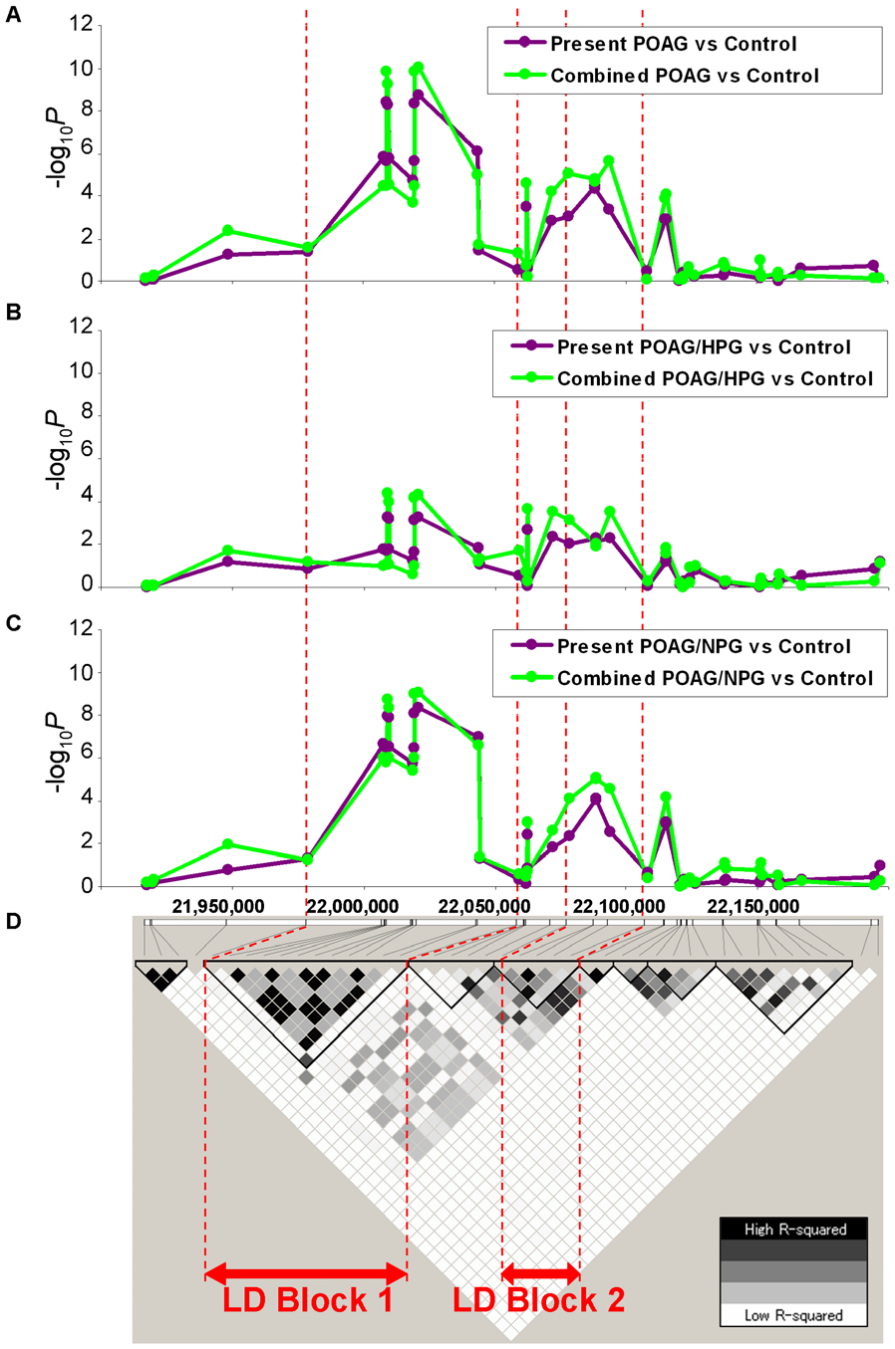
Combined analysis of the Present and Previous GWAS data in 9p21.3 locus. Three graphs representing the results of the association studies for POAG (A), HPG (B), and NPG (C) are shown in order from upper to lower, respectively. The results derived from the Present GWAS alone or the two combined GWAS data sets are indicated by the purple or green line graph plots, respectively. The lowest plot (D) shows an LD plot in the same region. All of the Present and Previous GWAS subject data was applied to draw the LD plot by Haploview v4.2. The color indicating pairwise *r* ^2^ and LD block were lined by using the confidence interval (95%) of pairwise *D′*. Vertical dashed redline indicates the same region between the upper graphs and LD Block 1 and 2 at the lowest.

Next, we updated the case and control groups of the previous GWAS population [10] with the latest clinical information and ended up with 411 POAG patients and 289 controls (hereafter referred to as “Previous GWAS”; Figure 1A). One SNP (rs8181047) of 5 genome-wide significant SNPs in *CDKN2B-AS1* identified from the Present GWAS was not able to be assessed in the Previous GWAS because a DNA probe of that particular SNP was not designed for the Affymetrix 500 K platform. Consequently, we combined the two sets of GWAS genotype data for the remaining 4 SNPs by use of the Mantel-Haenszel test [19], and the level of significance of all 4 SNPs increased from *P*<5.2×10^−9^ to *P*<5.8×10^−10^ (Table 1). These 4 SNPs were highly correlated with one another (r^2^>0.98), and were considered to have a similar effect for POAG based on the conditional analysis. These results suggested that it was effective to combine the data since the population stratification between the two data sets seemed to be ignorable, which was also supported by the results of the heterogeneity test. Moreover, we assessed the confounding effects for these SNPs with respect to age and gender, and none were found to be statistically significant (Table S2), thus suggesting that the obtained association results were specific to the case-control comparison. In addition, the two sets of GWAS data for 40 SNPs surrounding the significant 4 SNPs in the 9p21.3 locus were also combined and analyzed (Table S3, Table S4). Since these 44 SNPs, in total, were genotyped in both the Present and Previous GWAS, and all of the SNPs passed the genotyping quality controls, these SNPs should prove useful in providing a broader overview of the significance of the locus. The results showed that the significance of combined SNPs was generally high between 22.0–22.1 Mb (Figure 3A). Within the particular locus, there seemed to be two distinct LD blocks; one in the side including genome-wide significant SNPs (“LD Block 1” in Figure 3D) and one in the other side with modestly associated SNPs (“LD Block 2” in Figure 3D).

**Table 1:** Association results of genome-wide significant SNPs in the Present GWAS.

			Previous GWAS			Present GWAS			Previous & Present Combined		
SNP:risk allele	Chr.	Position	Frequency^a^	*P*	OR (95% CI)	Frequency^a^	*P*	OR (95% CI)	*P* b	OR (95% CI)	Het-*P* c
rs523096:A	9	22,009,129	0.89/0.84	6.0×10^−3^	1.56 (1.13–2.14)	0.89/0.82	3.8×10^−9^	1.86 (1.51–2.29)	1.6×10^−10^	1.76 (1.48–2.10)	0.42
rs518394:C	9	22,009,673	0.89/0.85	1.5×10^−2^	1.49 (1.08–2.07)	0.89/0.82	5.2×10^−9^	1.85 (1.50–2.28)	5.8×10^−10^	1.74 (1.46–2.07)	0.34
rs564398:A	9	22,019,547	0.90/0.85	4.7×10^−3^	1.58 (1.15–2.18)	0.90/0.82	4.6×10^−9^	1.86 (1.51–2.29)	1.4×10^−10^	1.77 (1.49–2.11)	0.47
rs7865618:A	9	22,021,005	0.90/0.85	6.3×10^−3^	1.56 (1.13–2.14)	0.90/0.82	2.0×10^−9^	1.88 (1.53–2.32)	9.0×10^−11^	1.78 (1.50–2.12)	0.39

a Risk allele frequency in POAG/controls.

b *P* value of combined 2 GWAS results by Mantel-Haenszel test.

c *P* value of Cochran's Q heterogeneity test between Previous and Present GWAS.

For the subtype analyses (Figure 1B), the POAG patients from both the Present and Previous GWAS populations were divided into two subtypes based on the clinical record of IOP measurement: 1) POAG patients with HPG (IOP≥22 mmHg) and 2) those with NPG (i.e., patients who consistently showed an IOP of less than 22 mmHg) [3]. The number of subjects in each subtype was found to be 330 HPG and 503 NPG patients and 215 HPG and 196 NPG patients in the Present and Previous GWAS, respectively (Figure 1B). When we performed the analysis separately using the Present GWAS data set, the Q-Q plots of HPG vs control and NPG vs control appeared to be quite different from one another (Figure S1B, C). In particular, the HPG results indicated non-deviated Q-Q plots (Figure S1B). In fact, although none of the SNPs were genome-wide significant for HPG (Figure S4A), we obtained 4 genome-wide significant SNPs that passed the Bonferroni correction threshold for NPG (Table S1, Figure S4B). As for NPG, the significance level of all 4 SNPs also increased when the two data sets were combined (Figure 3C, Table S3, Table S4), however, the significance was still far from the Bonferroni threshold of significance in relation to HPG (Figure 3B, Table S3, Table S4). Although the significance level of SNPs residing in LD Block 2 showed slight differences among the different subtypes, the level in LD Block 1 seemed to be determining the difference of significance between the two subtypes, thus suggesting that the variants on LD Block 1 are closely associated with the susceptibility to glaucoma in the 9p21.3 locus (Figure 3D). Surprisingly, all of the significant SNPs from NPG overlapped with those obtained from POAG (Table S1). However, when we performed the heterogeneity test between the HPG and NPG groups, the results were not significant (*P* = 0.21–0.25, Table S3), suggesting that the significant differences between the two subtypes could be due to the small sample size of the HPG group. Although we need to confirm the above results using a larger HPG cohort, these results suggested that the significant SNPs identified in *CDKN2B-AS1* on the 9p21.3 locus probably serve as genetic markers of glaucoma, which could be useful for investigating the etiology of glaucoma with respect to the vulnerability of the optic nerve to IOP.

## Discussion

In this study, we successfully identified genetic markers in *CDKN2B-AS1* strongly associated with POAG and POAG/NPG, but not with HPG, by analyzing two GWAS data sets using independent study populations totaling 2,219 Japanese subjects. For the case-control study of the Present GWAS, we excluded the samples without hesitation that didn't meet our strict quality control (see Supporting Information S1). Since we succeeded in excluding the samples possessing a fair amount of no-call or missed-call genotype data, our more stringent filters certainly improved the actual genotyping results for the association studies performed later. Thus, we were able to obtain an increased level of significance without any substructure effects of the two populations based on the definite diagnosis when combining the genotyping results of the Present and Previous GWAS data sets (Figure 3).

Using our polished data sets derived from distinct case and control subjects (see Methods and Supporting Information S1), we succeeded in identifying a cluster of genome-wide significant SNPs associated with POAG in *CDKN2B-AS1*, a non-coding gene with an unknown function, on chromosome 9p21.3 (Figure 2A, B). Several variants in chromosome 9p21 have been found to be associated with a variety of common diseases, and 9p21 was initially identified as a locus for coronary artery disease (CAD) [20]–[23] and type-2 diabetes [24]–[26]. However, little is known about the biological meanings underlying the locus, as 9p21 is a “gene desert” locus and most of the variants identified were non-coding. Recently, Harismendy et al. [27] reported that they have identified 33 enhancers in 9p21, some of which being within *CDKN2B-AS1*, and it turned out to be the second densest gene desert for predicted enhancers, thus suggesting the regulatory role of sequences residing within non-coding loci. They finally determined a few adjacent, as well as distant (>45 kb), target gene regions relevant to CAD biology physically interacting with the enhancer by 3D-DSL (also known as “4C”), a chromatin conformation capture technology, in human vascular endothelial cells. Overall, their study has provided an excellent example of a solution to link the unknown meanings of statistical association to a biological function. Since the POAG variants were also identified in *CDKN2B-AS1*, the variants would probably affect the expression level of the downstream genes *CDKN2A* and *CDKN2B*. In fact, Burdon et al. reported up-regulated *CDKN2A* and *CDKN2B* expression in response to the elevated IOP [14], suggesting the involvement of the locus in molecular pathways leading to glaucoma development. Moreover, the possibility that the variants would also affect the distant unidentified target genes in the context of the complex etiology of glaucoma, as well as the nature of the identified locus [27], cannot be ruled out.

To date, several institutions have attempted to discover variants for glaucoma by conducting a GWAS, and a few published studies have reported the association of particular loci with POAG using Caucasian subjects (Table S5). Thorleifsson et al. reported the association of common variants near *CAV1*/*CAV2* on 7q31.1 using a population of European ancestry [13]. In contrast, Kuehn et al. reported that they failed to replicate their results in a United States cohort [15]. Moreover, meta-analysis of the association results derived from several institutions of Northern Europe, including the data from Thorleifsson et al. [13], identified a few new loci associated with POAG, including the *CDKN2B-AS1* locus on 9p21.3 [17]. Recently, the new loci at *TMCO1* on 1q24.1 and *CDKN2B-AS1* were reported to be associated with Australian populations [14]. Thus far, only the association of the *CDKN2B-AS1* locus was replicated, even within the same ethnicity of European descent. Interestingly, Thorleifsson et al. also showed a modest association with the *CAV1*/*CAV2* locus using Chinese subjects [13], although the allele frequency of the particular variant in Asian subjects was quite low when compared with that in Caucasians, suggesting etiological differences due to the genetic background. In fact, according to the results of our Present GWAS for POAG, we were unable to replicate the association with the *CAV1*/*CAV2* and *TMCO1* loci (Figure S5F, G, Table S5). Moreover, in the Present GWAS, we were unable to replicate the association results of the 6 SNPs identified in the previous study [10] (Table S5), even though the populations were of the same ethnic background, thus suggesting that we still need to discover authentic variants, irrespective of the difference in ethnicities, to elucidate the complex etiology of glaucoma. Consequently, it should be noted that the variants identified in the *CDKN2B-AS1* locus in this study using a Japanese population seemed to be shared with the Caucasian subjects (Table S5), thus showing that we have successfully obtained one of the authentic variants for POAG that is not ethnicity related.

In this study, we also subdivided the POAG subjects into two subtypes based on the IOP level measurements in an attempt to discover subtype-specific variants, which would be useful for investigating the different mechanism of each pathogenesis. Since the clinical states of both subtypes overlap almost completely, they are usually categorized as a single disease. However, as a unique epidemiological distribution compared with other ethnic groups, ∼92% of the Japanese POAG patients fall into the NPG category, as determined by the Tajimi study [4], a robust epidemiology study. In fact, another Japanese group performed a GWAS focused on discovering NPG-specific variants [12]. They reported that the SNPs in *SRBD1* on 7q31.1 and *ELOVL5* on 6p12.1 were associated with NPG, although our group was unable to replicate those results (Figure S5D, E). On the contrary, we obtained an unexpected result that the variants associated with NPG were completely identical to those associated with POAG in *CDKN2B-AS1* identified in this study (Table S1). In contrast, none of the variants were significant for HPG (Figure 3B). Although we cannot rule out that the significance could be due to the differences in sample size between HPG and NPG cases according to the heterogeneity test (*P* = 0.21–0.25; Table S3), the results suggested that the genetic loci identified are most likely components of the molecular mechanism specific for NPG. It has been reported that there are differences in vulnerability of the optic nerve to IOP between HPG and NPG [18]. It has also been theorized that *CDKN2B-AS1* affects the susceptibility of the optic nerve [14], [28]. Since the variants identified in this study seemed to be shared among different ethnicities in functional aspects as well, the other variants should be contributing to the unique epidemiology of NPG in Japanese. By continuing to build upon the detailed investigation, such as obtaining in-depth sequencing data of the non-coding 9p21 locus, it might be possible to reveal not only the molecular mechanism of glaucoma pathogenesis but also the genetic diversity that resides within the locus among different ethnic backgrounds.

## Methods

### Ethics statement

This study was approved by the Institutional Review Board of Kyoto Prefectural University of Medicine and all procedures were conducted in accordance with the Declaration of Helsinki. All participants provided written informed consent after an explanation of the nature and possible consequences of the study.

### Case and control subjects for the Present GWAS

All participants were interviewed to determine their familial history of glaucoma and other ocular or general diseases. A total of 2,126 Japanese participants, including POAG patients, healthy volunteers, and patients with other ocular diseases, were recruited between March 2005 and December 2010 at the University Hospital of Kyoto Prefectural University of Medicine (Kyoto, Japan) to give peripheral blood samples for this study. A third person who was blind to both the blood sampling and genotyping assigned an anonymous code to each blood sample. Genomic DNA used for the genotyping experiments was isolated from the blood, and Epstein-Barr virus (EBV)-transformed lymphocytes were prepared from the remaining blood as previously described [29] to serve as the future resource of genomic DNA. POAG patients and controls suitable for this study were precisely selected based on the strict diagnosis as previously described [10]. In particular, subtype selection was performed by dividing POAG into two categories according to peak IOP without treatment; POAG/High Pressure Glaucoma (POAG/HPG, defined as ≥22 mmHg) and POAG/Normal Pressure Glaucoma (POAG/NPG) [3]. All of the diagnostic procedures, including case-control selection and subtype classification, were performed by three ophthalmologists (YI, MU, and KM) from a single institution. The age and female/male ratio of all of the subjects used in the Present and Previous GWAS are shown in Table S6. To examine the possible confounding effects of age and gender, we assessed the correlations between the values and the genotype data from the case and control samples by one-way ANOVA or chi-square test (Table S2).

### SNP genotyping and quality control for the Present GWAS

First, 906,600 SNPs were genotyped for 2,126 Japanese subjects by Genome-Wide Human SNP Array 6.0 (Affymetrix, Santa Clara, CA) according to the manufacturer's instructions. As for the Present GWAS population, 839 POAG patients and 708 controls were selected after performing the quality control (QC) as described (Supporting Information S1). To exclude the potential inclusion of genetically related subjects into the population, identity-by-descent (IBD) estimates were performed for all possible combinations by PLINK v1.07 (http://pngu.mgh.harvard.edu/~purcell/plink/). In total, 6 POAG patients and 22 controls were assumed to be in first-degree relationships or in relationships with a few more-distant relatives, and were thus excluded from the Present GWAS population. Finally, the Present GWAS population ended up with 833 POAG patients, comprised of 330 HPG and 503 NPG patients, and 686 controls. Population stratification for the present GWAS population was examined by principal component analysis using EIGENSTRAT software v3.0 (http://genepath.med.harvard.edu/~reich/Software.htm). As for the reference genomic population, the four HapMap populations (CEU, YRI, JPT, and CHB) were simultaneously applied to EIGENSTRAT. The generated cluster plots indicated that our POAG and control population was genetically clustered within the JPT population, and there was no outlier sample (Figure S6). We performed SNP quality control for the population on the autosomal SNPs based on the following QC filters: (i) call rate per SNPs in case and control samples ≥95%, (ii) minor allele frequency (MAF) in case and control samples ≥1%, and (iii) Hardy-Weinberg equilibrium (HWE) in control samples P≥0.001. Consequently, we analyzed the remaining 653,519 SNPs for the Present GWAS population.

### Population update for reanalyzing the Previous GWAS

Since 6 years had passed since the patients and healthy volunteers who participated in our Previous GWAS [10] were first recruited, we updated the case (n = 418) and control (n = 300) groups based on the latest clinical information. In total, 7 samples from the case group and 11 samples from the healthy control group were removed. The population for the reanalysis finally ended up with 411 POAG patients, comprised of 215 HPG and 196 NPG patients, and 289 controls. The Previous GWAS data was obtained by GeneChip® Human Mapping 500 K Array platform (Affymetrix) containing 500,568 SNPs. The quality control for the reanalysis was performed by using the same QC filters as used in the Present GWAS. Genotype data derived from the particular locus for combining with the Present GWAS data was extracted from these filtered SNPs.

### Data management and statistical analysis

To manage and analyze all of the genotype data, we used our in-house Genoika Server System (SASA Plus Co., Ltd., Fukuoka, Japan), which was well improved from the system used in the Previous GWAS [10], [30] by the same system engineers. In addition, to manage the Previous GWAS data effectively, the Labo Server System (World Fusion Co., Ltd.) was used simultaneously. The Genoika Server System comes with PLINK v1.07 (http://pngu.mgh.harvard.edu/~purcell/plink/), the R program v 2.9.2 and 2.10.1 (http://www.r-project.org/), EIGENSTRAT software v3.0 (http://genepath.med.harvard.edu/~reich/Software.htm), and Haploview 4.2 (http://www.broadinstitute.org/scientific-community/science/programs/medical-and-population-genetics/haploview/haploview) built in, and all the analyses were performed by use of this system. In addition, Microsoft Office Excel 2003 (Microsoft Corporation, Redmond, WA) was used for preparing the data sets and statistical analysis. The frequency of alleles in the case and control samples was compared by use of the basic allele test. The odds ratio (OR) and the upper and lower limit of the 95% confidence interval (CI) of each SNP were calculated for the allele possessing a higher frequency in the case samples than in the control samples. The HWE was evaluated by the chi-square test. Q-Q plots were generated by ranking the observed values from minimum to maximum and plotting them against their expected chi-square values using the “snpMatrix” package ver 1.14.6 in the R program (http://www.r-project.org/). We applied the Mantel-Haenszel test [19] to combine the data derived from the two data sets in order to reduce potential negative effects arising from the biases in age and female/male ratio among the populations (Table S6).

## Supporting Information

Figure S1 **Q-Q plots for the Present GWAS.** Quantile-quantile (Q-Q) plots for the Present GWAS of POAG (A), HPG (B), and NPG (C) were generated by ranking the observed chi-square values from minimum to maximum and plotting them against their expected values. Genomic inflation factors (λ) are also shown. These plots were created using the R-package snpMatrix.
(PDF)

Figure S2 **LD plots in the 9p21 locus.** LD plots were generated from the Present GWAS data. The SNPs applied to these plots and the span of the region are the same as shown in Figure 2B. Upper and lower plots indicate the value of pairwise D′ and r2, respectively. The positions of 5 SNPs, which passed the Bonferroni correction threshold in the Present GWAS, are drawn in vertical dashed lines. These LD plots were generated using Haploview v4.2.
(PDF)

Figure S3 **Genotyping error check.** 2D-cluster plots for the representative SNPs obtained from chromosome 9 (A), chromosome 1 (B), chromosome 5 (C), and chromosome 12 (D) that showed genome-wide significance. These 2D cluster plots were drawn by Genotyping Console 4.1 software (Affymetrix).
(PDF)

Figure S4 **GWAS results of two subtypes.** Association results of the Present GWAS for the two subtypes of POAG, HPG (A) and NPG (B). The horizontal dashed line in each plot indicates the Bonferroni correction threshold for each study.
(PDF)

Figure S5 **Evaluation of previously reported loci/genes.** Evaluation of the POAG-associated loci/genes reported previously using our Present GWAS data. The genes located in the loci are as the follows: (A) ZP4, (B) PLXDC2, (C) TMTC2, (D) SRBD1, (E) ELOVL5, (F) CAV1/CAV2, and (G) TMCO1. Each locus was evaluated by the results of POAG vs control, POAG/HPG vs control, and POAG/NPG vs control. The plotted SNPs were selected from QC filtered SNPs. Target genes are placed in the center of the 500-kb spanned region. Plots are shown with genomic annotation and HapMap LD (JPT+CHB) made by the UCSC Genome Browser (Human Mar. 2006).
(PDF)

Figure S6 **Population stratification analysis.** Population stratification analysis by EIGENSTRAT in the Present GWAS data set. POAG samples separated with HPG and NPG groups were applied. The version of HapMap reference data is release22 (Build36). Since Control, NPG, HPG, and HapMap JPT samples were tightly overlapped, only the plots for NPG (yellow cross) are visible.
(PDF)

Table S1 **Genome-wide significant SNPs that passed the Bonferroni correction threshold in the Present GWAS.**
(PDF)

Table S2 **Analysis of confounding effects of age and sex for the candidate SNPs.**
(PDF)

Table S3 **Present GWAS results in 9p21.3 locus.**
(PDF)

Table S4 **Combined GWAS results in 9p21.3 locus.**
(PDF)

Table S5 **Association results of Present GWAS for previously reported SNPs.**
(PDF)

Table S6 **Characteristics of the samples.**
(PDF)

Supporting Information S1 **Quality control for genotyping.**
(PDF)
