# Supplementary figures and images for: Common Variants in CDKN2B-AS1 Associated with Optic-Nerve Vulnerability of Glaucoma Identified by Genome-Wide Association Studies in Japanese
Source: PLoS One. 2012 Mar 12;7(3):e33389. doi: 10.1371/journal.pone.0033389 (PMC3299784; doi:10.1371/journal.pone.0033389)

Figure S1

A

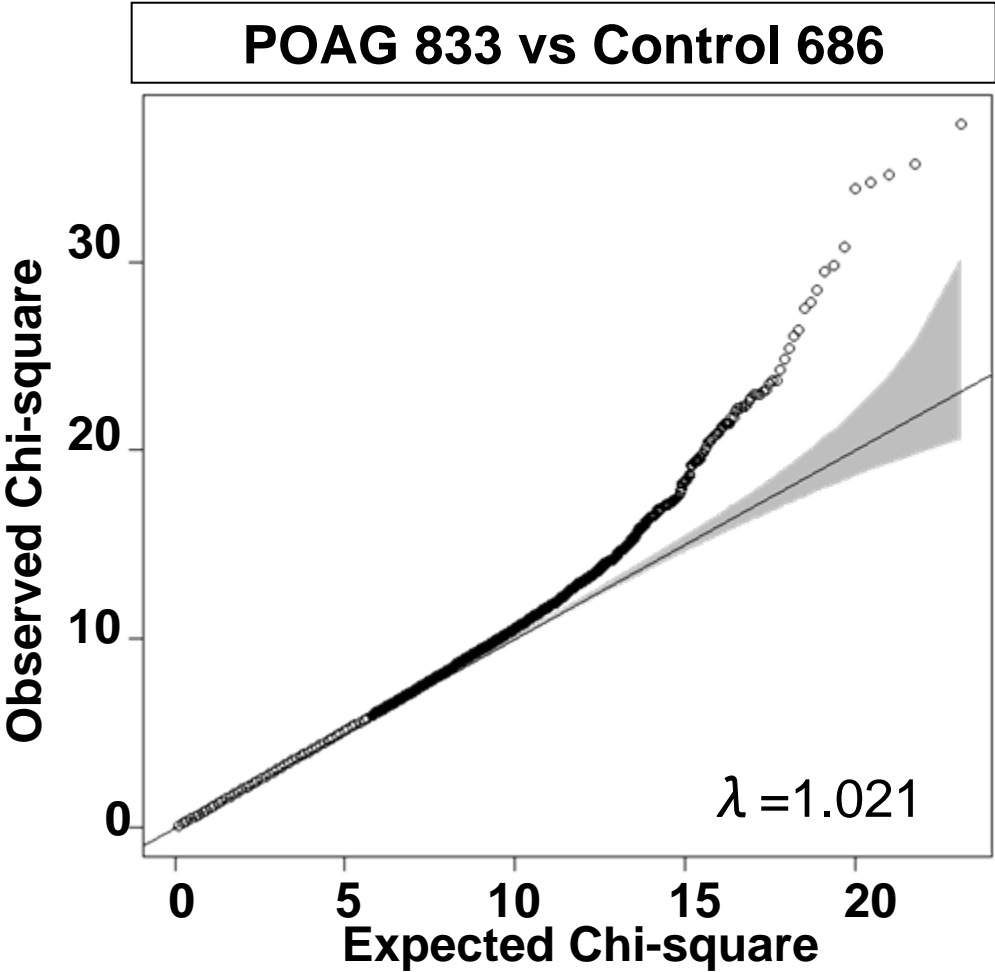

B

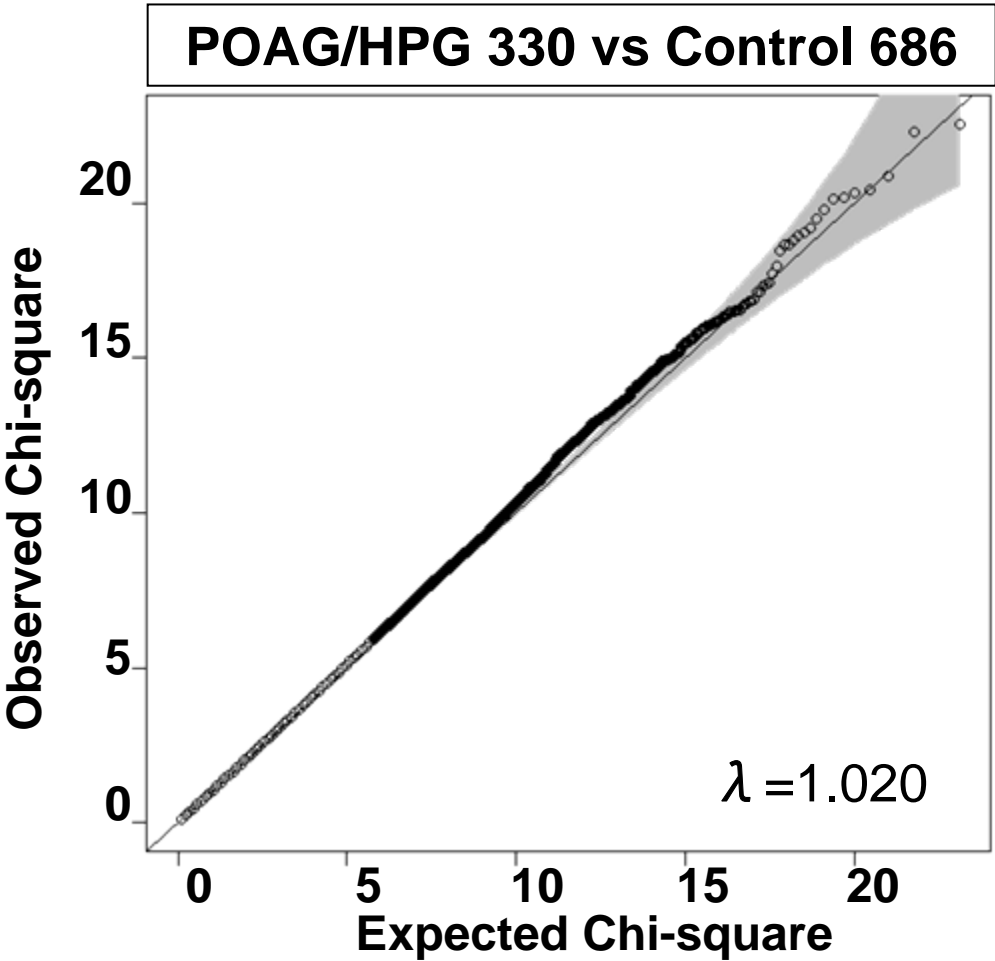

**C**

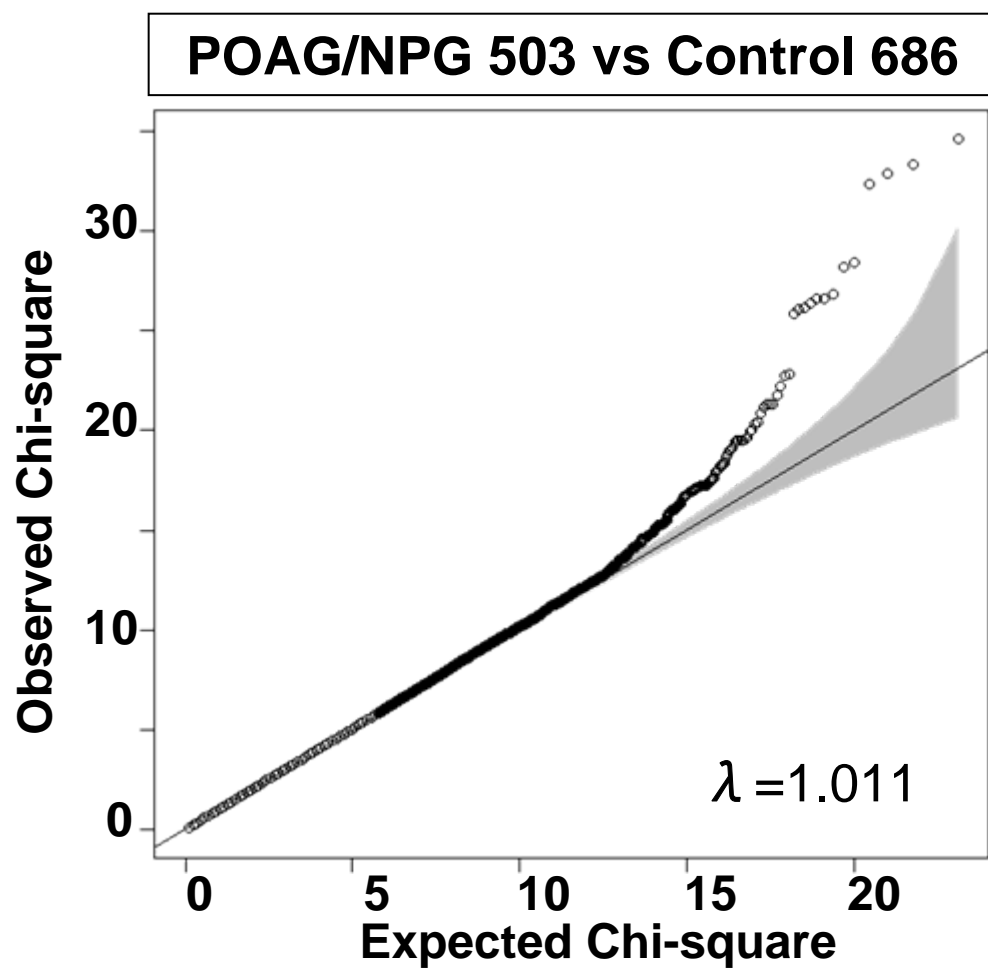

Supplement: Figure S1 — Q-Q plots for the Present GWAS. Quantile-quantile (Q-Q) plots for the Present GWAS of POAG (A), HPG (B), and NPG (C) were generated by ranking the observed chi-square values from minimum to maximum and plotting them against their expected values. Genomic inflation factors (λ) are also shown. These plots were created using the R-package snpMatrix. (PDF) [file pone.0033389.s001.pdf]

Figure S2

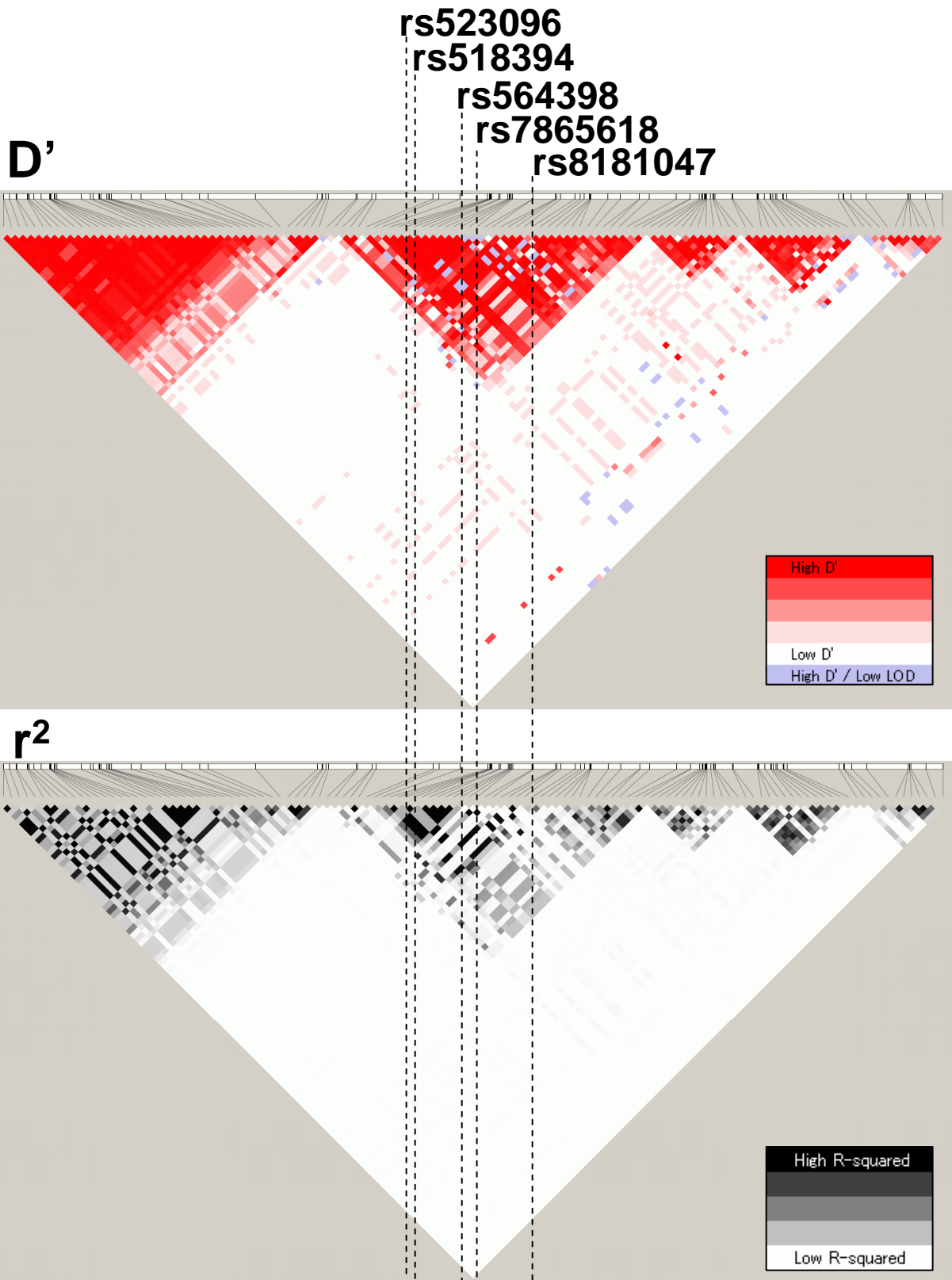

Supplement: Figure S2 — LD plots in the 9p21 locus. LD plots were generated from the Present GWAS data. The SNPs applied to these plots and the span of the region are the same as shown in Figure 2B. Upper and lower plots indicate the value of pairwise D′ and r2, respectively. The positions of 5 SNPs, which passed the Bonferroni correction threshold in the Present GWAS, are drawn in vertical dashed lines. These LD plots were generated using Haploview v4.2. (PDF) [file pone.0033389.s002.pdf]

Figure S3

**A** rs7865618

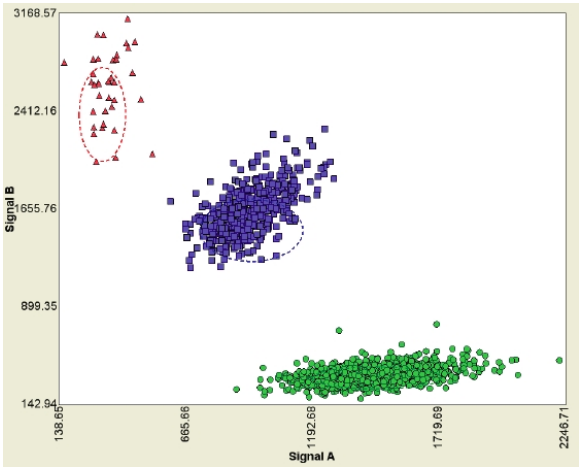

**B** rs6689160

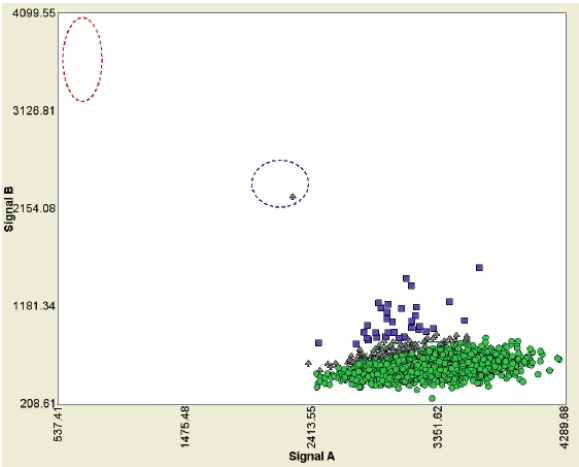

**C** rs41524744

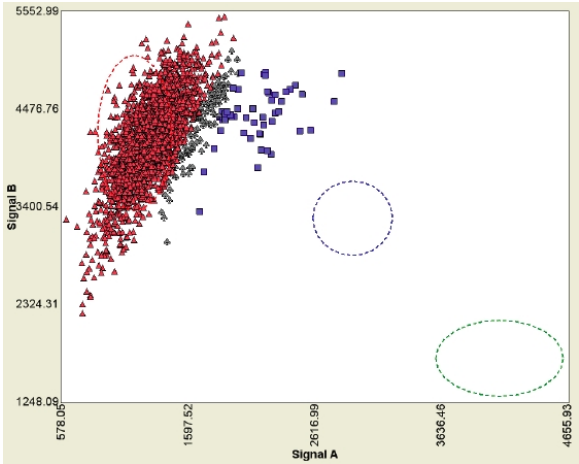

**D** rs1239904

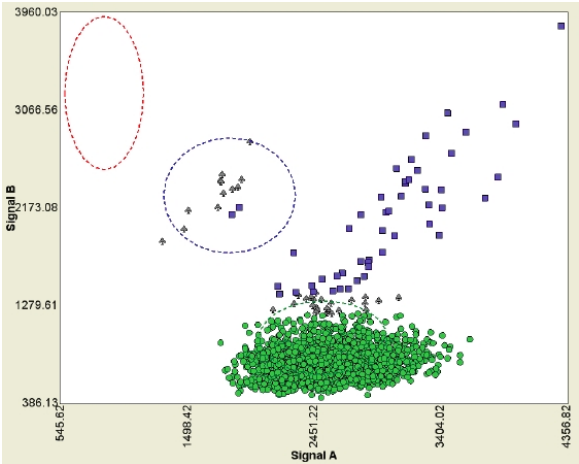

Supplement: Figure S3 — Genotyping error check. 2D-cluster plots for the representative SNPs obtained from chromosome 9 (A), chromosome 1 (B), chromosome 5 (C), and chromosome 12 (D) that showed genome-wide significance. These 2D cluster plots were drawn by Genotyping Console 4.1 software (Affymetrix). (PDF) [file pone.0033389.s003.pdf]

Figure S4

A

POAG/HPG 330 vs Control 686 : 653,317 SNPs

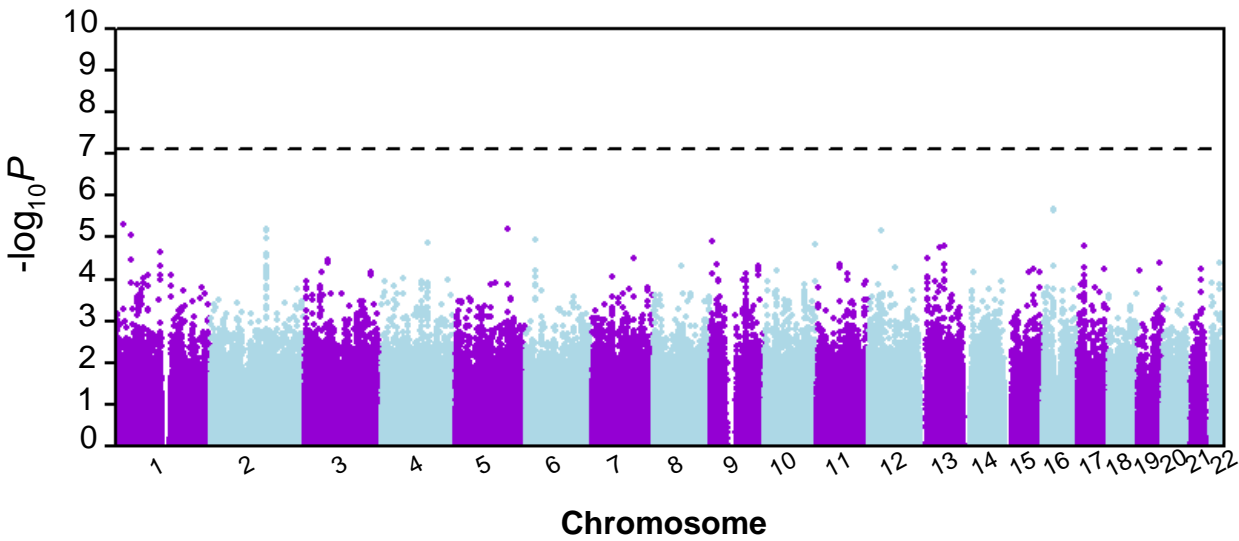

B

POAG/NPG 503 vs Control 686 : 654,200 SNPs

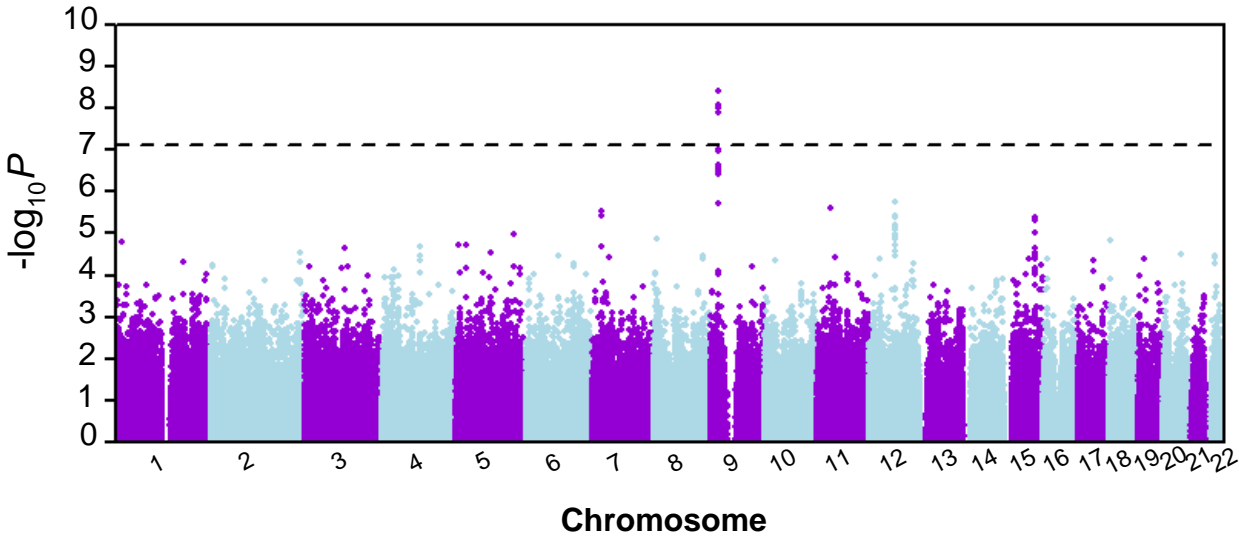

Supplement: Figure S4 — GWAS results of two subtypes. Association results of the Present GWAS for the two subtypes of POAG, HPG (A) and NPG (B). The horizontal dashed line in each plot indicates the Bonferroni correction threshold for each study. (PDF) [file pone.0033389.s004.pdf]

Figure S5

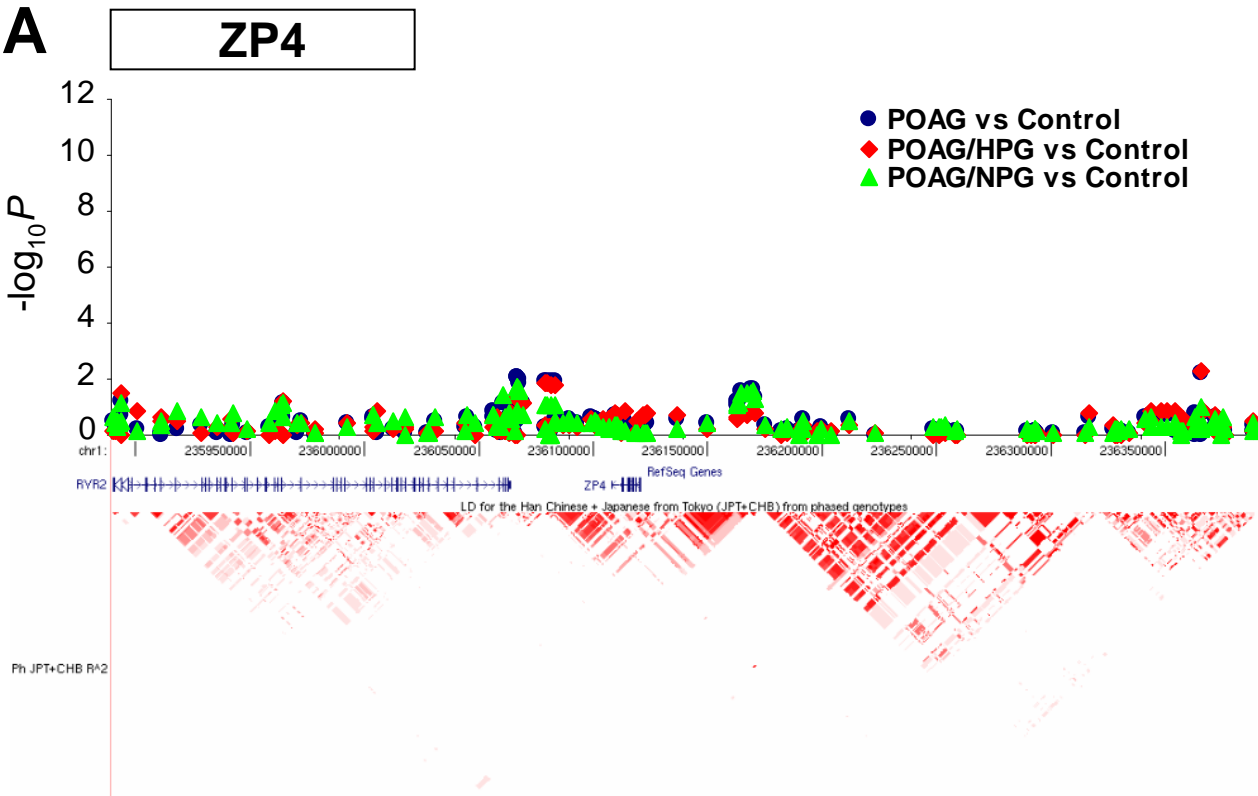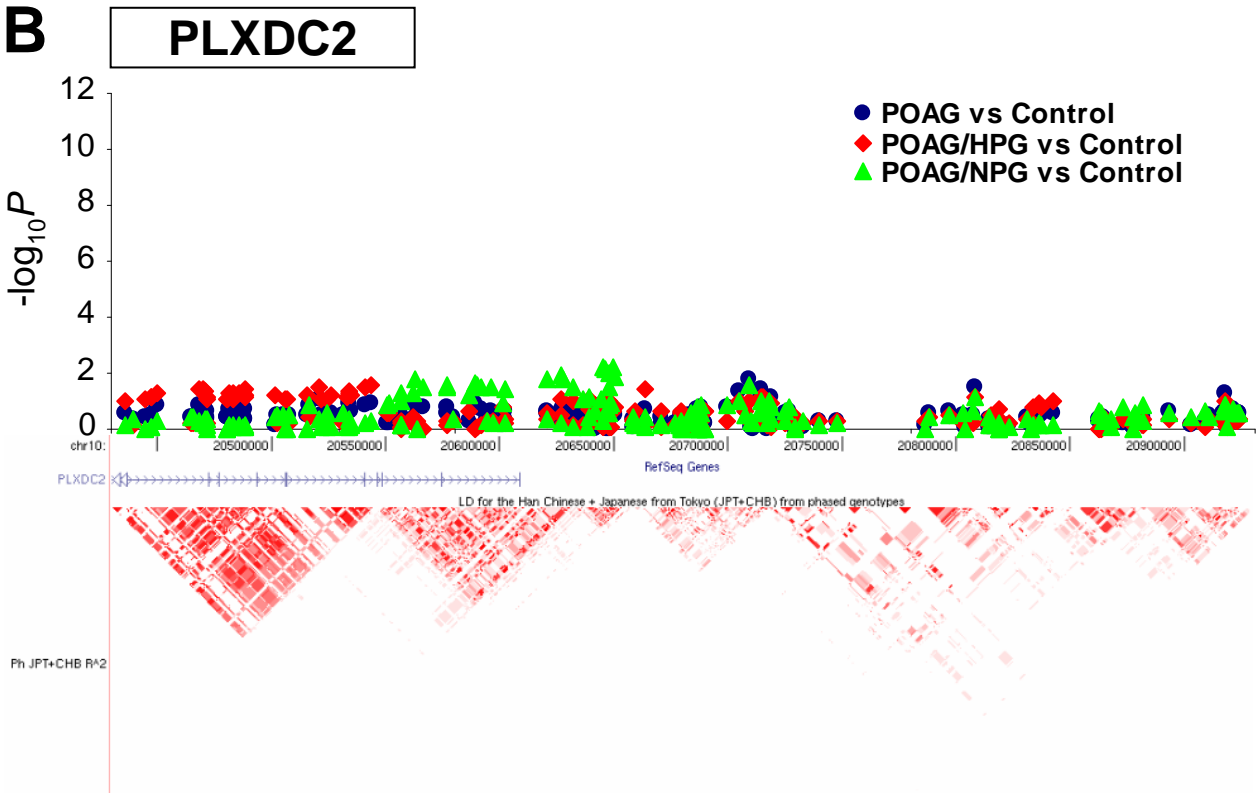

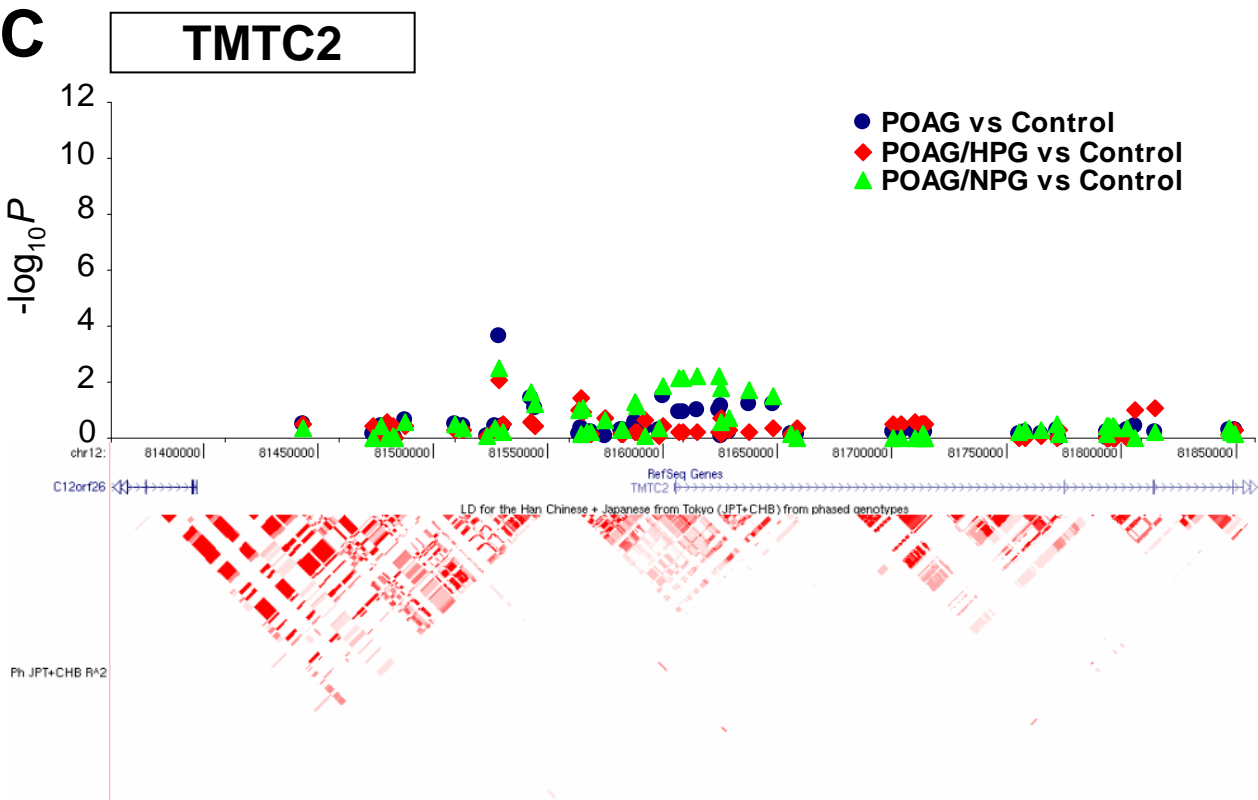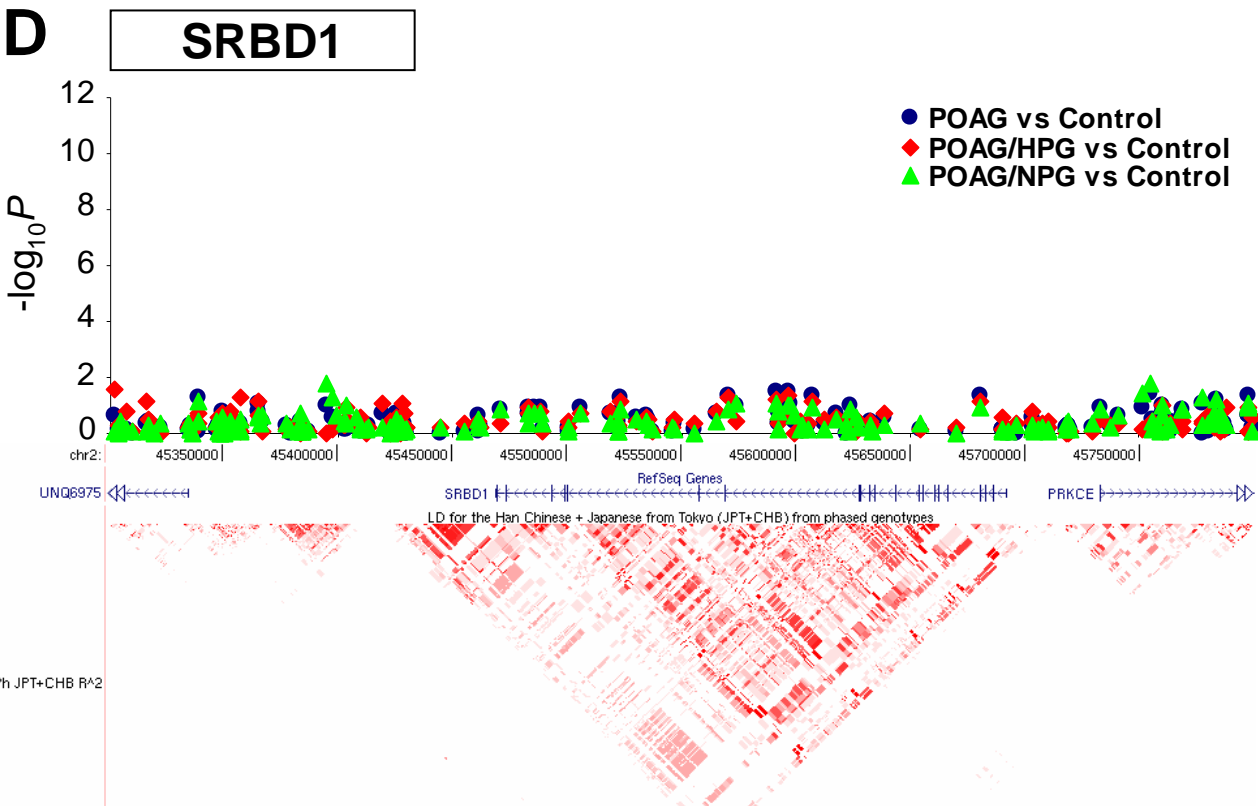

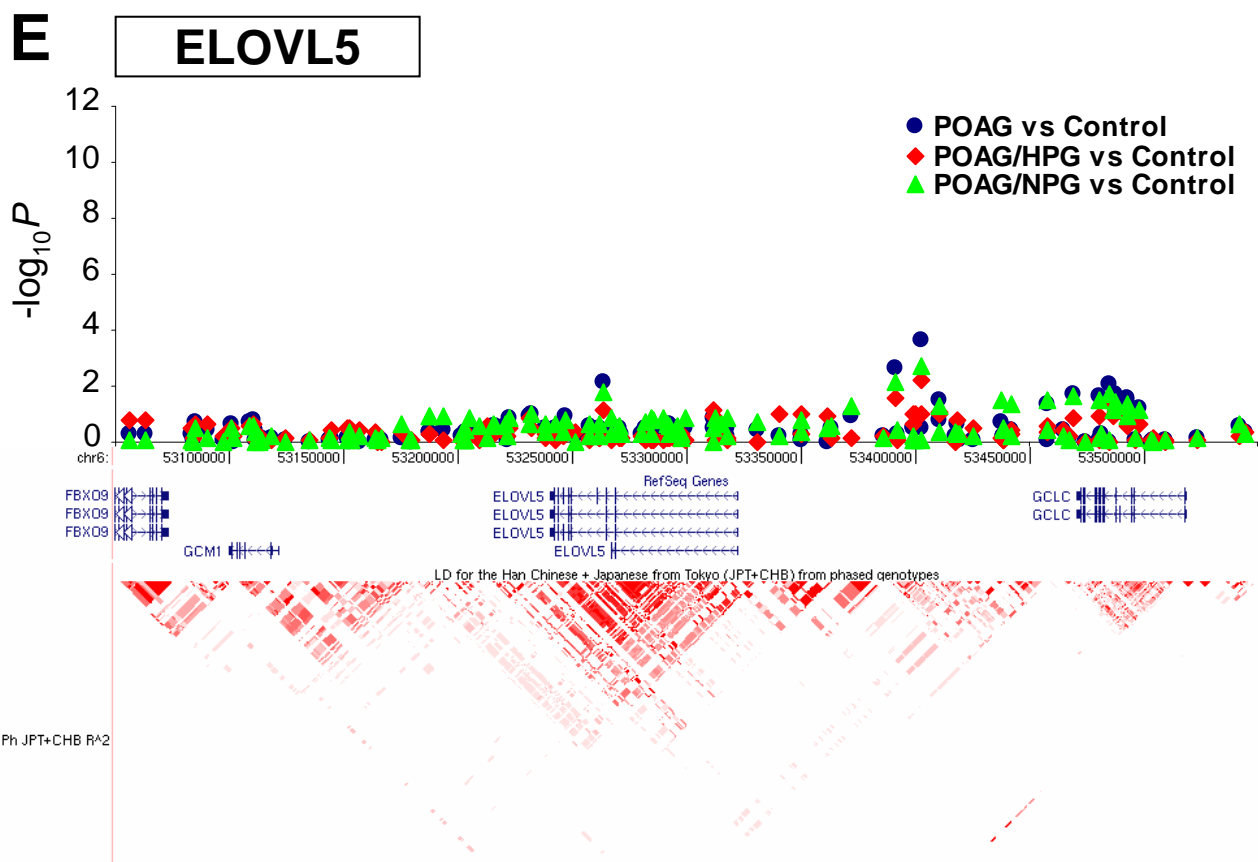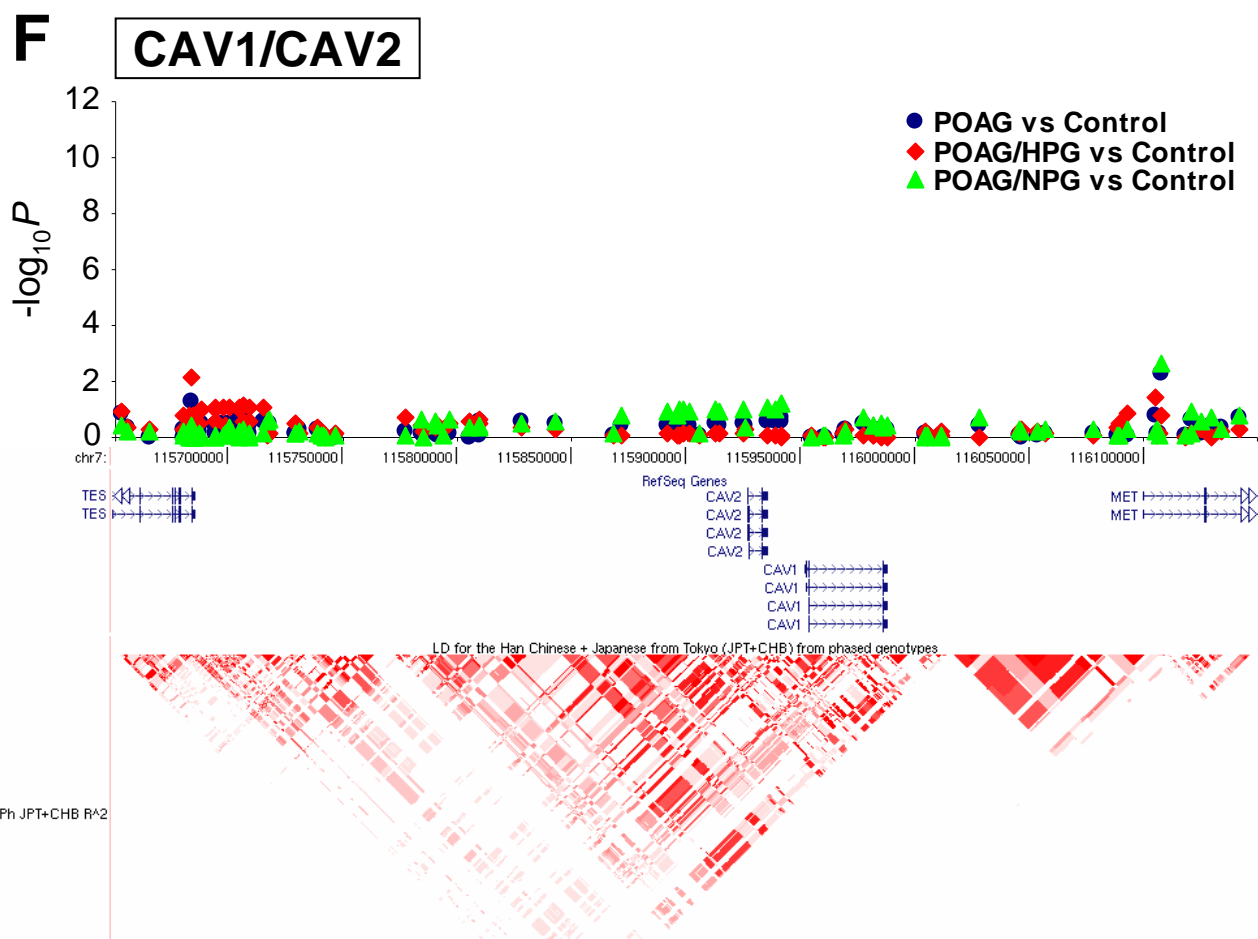

G

TMCO1

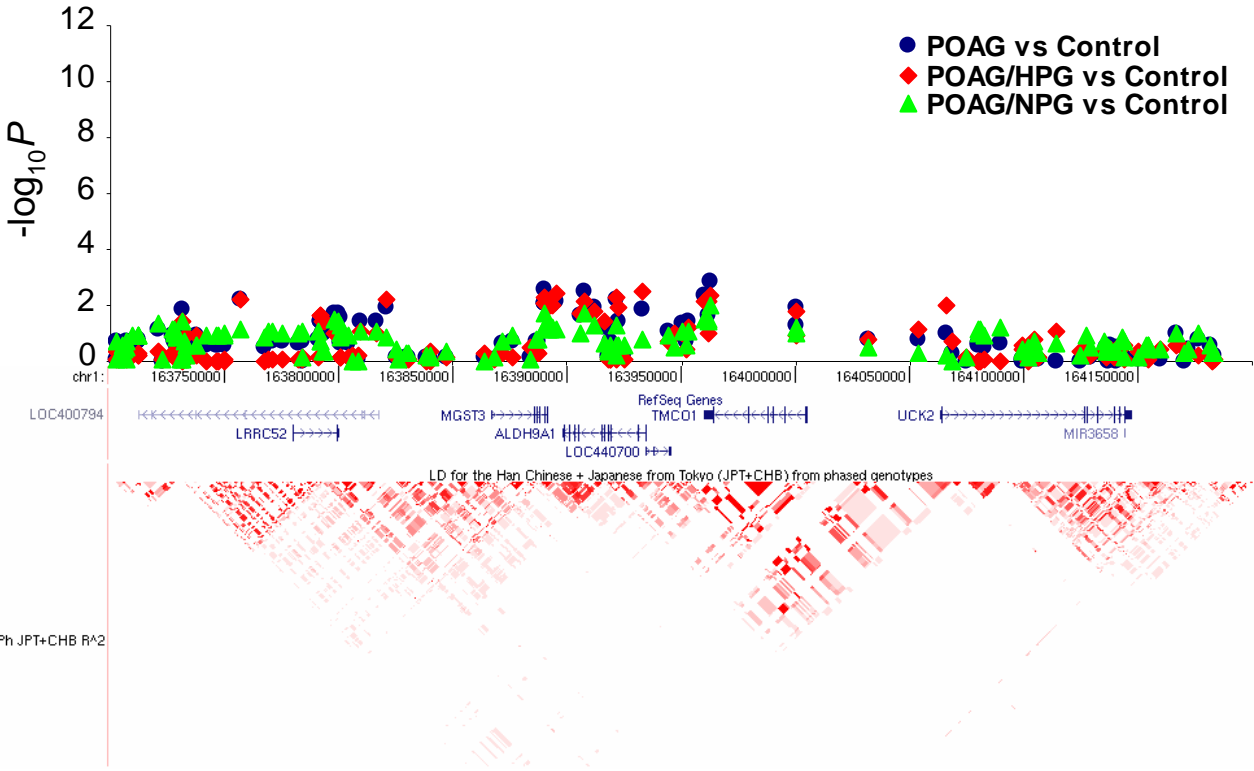

Supplement: Figure S5 — Evaluation of previously reported loci/genes. Evaluation of the POAG-associated loci/genes reported previously using our Present GWAS data. The genes located in the loci are as the follows: (A) ZP4, (B) PLXDC2, (C) TMTC2, (D) SRBD1, (E) ELOVL5, (F) CAV1/CAV2, and (G) TMCO1. Each locus was evaluated by the results of POAG vs control, POAG/HPG vs control, and POAG/NPG vs control. The plotted SNPs were selected from QC filtered SNPs. Target genes are placed in the center of the 500-kb spanned region. Plots are shown with genomic annotation and HapMap LD (JPT+CHB) made by the UCSC Genome Browser (Human Mar. 2006). (PDF) [file pone.0033389.s005.pdf]

Figure S6

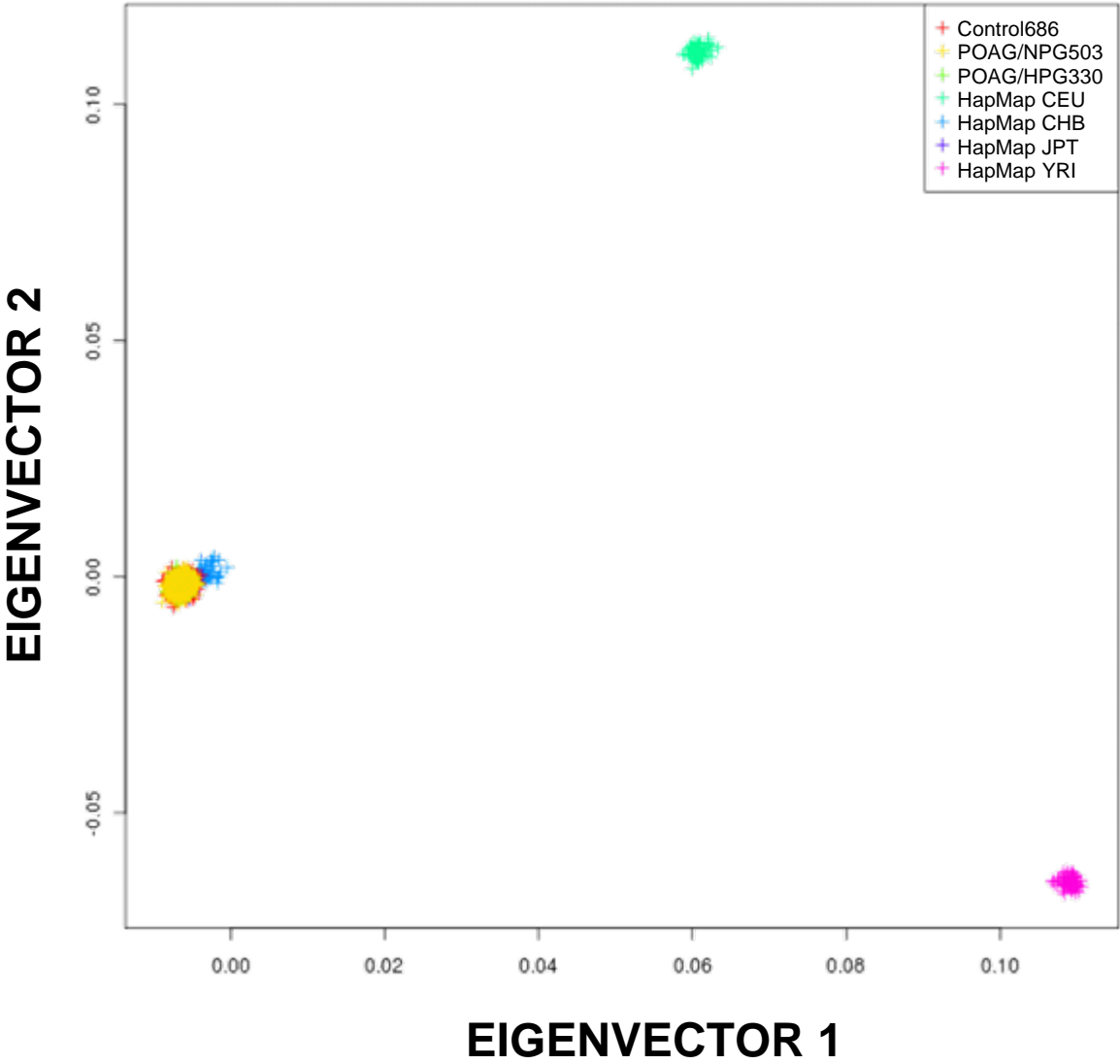

Supplement: Figure S6 — Population stratification analysis. Population stratification analysis by EIGENSTRAT in the Present GWAS data set. POAG samples separated with HPG and NPG groups were applied. The version of HapMap reference data is release22 (Build36). Since Control, NPG, HPG, and HapMap JPT samples were tightly overlapped, only the plots for NPG (yellow cross) are visible. (PDF) [file pone.0033389.s006.pdf]
