# Supplementary material for: Common Variants in CDKN2B-AS1 Associated with Optic-Nerve Vulnerability of Glaucoma Identified by Genome-Wide Association Studies in Japanese
Source: PLoS One. 2012 Mar 12;7(3):e33389. doi: 10.1371/journal.pone.0033389 (PMC3299784; doi:10.1371/journal.pone.0033389)
Supplement: Table S1 — Genome-wide significant SNPs that passed the Bonferroni correction threshold in the Present GWAS. (PDF) [file pone.0033389.s007.pdf]

Table S1

Study	Bonferroni correciton threshold[a]	SNP: risk allele	Chromosome	Position	Frequency[b]	*P*	OR (95% CI)
POAG vs Control	7.65×10^{-8} (0.05/653,519)						
		rs6689160:A	1q25.2	178,158,188	0.9988/0.9782	5.8×10^{-8}	17.88 (4.91-65.17)
		rs41524744:T	5p15.32	5,360,913	0.9981/0.9762	4.7×10^{-8}	12.98 (4.29-39.27)
		rs523096:A	9p21.3	22,009,129	0.8944/0.8200	3.8×10^{-9}	1.86 (1.51-2.29)
		rs518394:C	9p21.3	22,009,673	0.8944/0.8207	5.2×10^{-9}	1.85 (1.50-2.28)
		rs564398:A	9p21.3	22,019,547	0.8956/0.8219	4.6×10^{-9}	1.86 (1.51-2.29)
		rs7865618:A	9p21.3	22,021,005	0.8956/0.8200	2.0×10^{-9}	1.88 (1.53-2.32)
		rs8181047:G	9p21.3	22,054,465	0.8637/0.7837	6.5×10^{-9}	1.75 (1.45-2.12)
		rs1239904:A	12q15	68,726,913	0.9976/0.9747	3.0×10^{-8}	10.63 (3.97-28.45)
NPG vs Control	7.64×10^{-8} (0.05/654,200)						
		rs523096:A	9p21.3	22,009,129	0.9036/0.8200	1.0×10^{-8}	2.06 (1.60-2.64)
		rs518394:C	9p21.3	22,009,673	0.9036/0.8207	1.3×10^{-8}	2.05 (1.59-2.63)
		rs564398:A	9p21.3	22,019,547	0.9056/0.8219	8.3×10^{-9}	2.08 (1.61-2.67)
		rs7865618:A	9p21.3	22,021,005	0.9056/0.8200	4.2×10^{-9}	2.11 (1.64-2.71)
HPG vs Control	7.65×10^{-8} (0.05/653,317)						
		not applicable					

[a] Bonferroni correction threshold is set as (significance level 0.05) / (the number of filtered SNPs in each study).

[b] Risk allele frequency in POAG or NPG/controls.
