# Supplementary material for: Common Variants in CDKN2B-AS1 Associated with Optic-Nerve Vulnerability of Glaucoma Identified by Genome-Wide Association Studies in Japanese
Source: PLoS One. 2012 Mar 12;7(3):e33389. doi: 10.1371/journal.pone.0033389 (PMC3299784; doi:10.1371/journal.pone.0033389)
Supplement: Table S2 — Analysis of confounding effects of age and sex for the candidate SNPs. (PDF) [file pone.0033389.s008.pdf]

Table S2

SNP		POAG	POAG/HPG	POAG/NPG	Control
rs523096	sex[a]	0.2579	0.1513	0.3401	0.6873
	age[b]	0.8189	0.7255	0.9944	0.0999
rs518394	sex[a]	0.3159	0.2405	0.2874	0.6633
	age[b]	0.8606	0.5138	0.9548	0.4519
rs564398	sex[a]	0.2943	0.1623	0.3490	0.7913
	age[b]	0.8298	0.7434	0.9946	0.0662
rs7865618	sex[a]	0.2663	0.1598	0.3078	0.7196
	age[b]	0.8407	0.7430	0.9931	0.0966

[a] Data show the *P* value analyzed by 2×3 χ^2 test for male/female vs three genotypes.

[b] Data show the *P* value analyzed by one-way ANOVA for three genotypes.
