# Supplementary material for: Common Variants in CDKN2B-AS1 Associated with Optic-Nerve Vulnerability of Glaucoma Identified by Genome-Wide Association Studies in Japanese
Source: PLoS One. 2012 Mar 12;7(3):e33389. doi: 10.1371/journal.pone.0033389 (PMC3299784; doi:10.1371/journal.pone.0033389)
Supplement: Table S3 — Present GWAS results in 9p21.3 locus. (PDF) [file pone.0033389.s009.pdf]

Table S3

SNP	RA.[a]	Chr.	Position	POAG vs Control Freq.[b]	POAG vs Control P	POAG vs Control OR (95% CI)	POAG/HPG vs Control Freq.[b]	POAG/HPG vs Control P	POAG/HPG vs Control OR (95% CI)	POAG/NPG vs Control Freq.[b]	POAG/NPG vs Control P	POAG/NPG vs Control OR (95% CI)	HPG vs NPG *HetP*[c]
rs4977749	A	9	21,917,327	0.54/0.54	0.93	1.01 (0.87-1.16)	0.54/0.54	0.93	0.99 (0.82-1.20)	0.54/0.54	0.84	1.02 (0.86-1.20)	0.84
rs2518713	T	9	21,919,666	0.54/0.54	0.89	1.01 (0.88-1.17)	0.53/0.54	0.92	0.99 (0.82-1.19)	0.54/0.54	0.78	1.02 (0.87-1.20)	0.80
rs7864029	G	9	21,920,147	0.55/0.54	0.81	1.02 (0.88-1.18)	0.54/0.54	0.85	0.98 (0.81-1.18)	0.55/0.54	0.62	1.04 (0.88-1.23)	0.64
rs717326	G	9	21,948,524	0.11/0.09	0.06	1.27 (0.99-1.62)	0.11/0.09	0.06	1.34 (0.99-1.82)	0.10/0.09	0.16	1.22 (0.93-1.61)	0.69
rs3731201	G	9	21,978,896	0.02/0.01	0.04	2.04 (1.02-4.07)	0.02/0.01	0.14	1.91 (0.82-4.42)	0.02/0.01	0.05	2.12 (1.00-4.49)	0.88
rs643319	C	9	22,007,836	0.69/0.61	1.5×10^{-6}	1.45 (1.24-1.68)	0.66/0.61	0.02	1.26 (1.04-1.54)	0.71/0.61	2.2×10^{-7}	1.58 (1.33-1.88)	0.12
rs7044859	A	9	22,008,781	0.69/0.61	2.3×10^{-6}	1.44 (1.24-1.67)	0.66/0.61	0.02	1.26 (1.04-1.53)	0.71/0.61	3.7×10^{-7}	1.57 (1.32-1.87)	0.13
**rs523096**[d]	A	9	22,009,129	0.89/0.82	3.8×10^{-9}	1.86 (1.51-2.29)	0.88/0.82	5.2×10^{-4}	1.61 (1.23-2.12)	0.90/0.82	1.0×10^{-8}	2.06 (1.60-2.64)	0.25
**rs518394**[d]	C	9	22,009,673	0.89/0.82	5.2×10^{-9}	1.85 (1.50-2.28)	0.88/0.82	6.0×10^{-4}	1.61 (1.22-2.11)	0.90/0.82	1.3×10^{-8}	2.05 (1.59-2.63)	0.25
rs10757264	C	9	22,009,732	0.69/0.61	1.6×10^{-6}	1.44 (1.24-1.68)	0.67/0.61	0.02	1.27 (1.04-1.54)	0.71/0.61	2.8×10^{-7}	1.58 (1.32-1.88)	0.13
rs7049105	C	9	22,018,801	0.69/0.61	1.8×10^{-5}	1.39 (1.20-1.62)	0.66/0.61	0.06	1.21 (1.00-1.47)	0.71/0.61	1.9×10^{-6}	1.53 (1.28-1.82)	0.10
rs10965215	A	9	22,019,445	0.69/0.61	2.5×10^{-6}	1.44 (1.23-1.67)	0.67/0.61	0.02	1.25 (1.03-1.52)	0.71/0.61	3.3×10^{-7}	1.57 (1.32-1.87)	0.11
**rs564398**[d]	A	9	22,019,547	0.90/0.82	4.6×10^{-9}	1.86 (1.51-2.29)	0.88/0.82	7.5×10^{-4}	1.59 (1.21-2.09)	0.91/0.82	8.3×10^{-9}	2.08 (1.61-2.67)	0.21
**rs7865618**[d]	A	9	22,021,005	0.90/0.82	2.0×10^{-9}	1.88 (1.53-2.32)	0.88/0.82	5.2×10^{-4}	1.61 (1.23-2.12)	0.91/0.82	4.2×10^{-9}	2.11 (1.64-2.71)	0.21
rs10965219	C	9	22,043,687	0.70/0.61	8.3×10^{-7}	1.46 (1.26-1.70)	0.67/0.61	0.02	1.27 (1.05-1.55)	0.72/0.61	1.1×10^{-7}	1.60 (1.35-1.91)	0.11

rs17694572	T	9	22,044,356	0.02/0.01	0.04	2.15 (1.05-4.41)	0.02/0.01	0.09	2.09 (0.88-4.93)	0.02/0.01	0.05	2.20 (1.01-4.78)	0.94
rs16905599	T	9	22,059,144	0.06/0.06	0.31	1.17 (0.87-1.58)	0.07/0.06	0.31	1.22 (0.83-1.78)	0.06/0.06	0.46	1.14 (0.81-1.61)	0.81
rs12555547	G	9	22,062,040	0.93/0.93	0.53	1.10 (0.83-1.45)	0.94/0.93	0.45	1.16 (0.80-1.68)	0.93/0.93	0.73	1.06 (0.77-1.45)	0.73
rs9632884	C	9	22,062,301	0.72/0.66	3.4×10^{-4}	1.33 (1.14-1.55)	0.72/0.66	2.2×10^{-3}	1.38 (1.12-1.69)	0.71/0.66	4.1×10^{-3}	1.30 (1.09-1.55)	0.68
rs17761197	G	9	22,062,730	0.83/0.81	0.25	1.11 (0.93-1.34)	0.82/0.81	0.80	1.03 (0.81-1.31)	0.83/0.81	0.14	1.17 (0.95-1.45)	0.43
rs6475606	A	9	22,071,850	0.69/0.64	1.6×10^{-3}	1.28 (1.10-1.49)	0.70/0.64	4.5×10^{-3}	1.34 (1.09-1.63)	0.68/0.64	0.01	1.24 (1.04-1.47)	0.61
rs10757272	A	9	22,078,260	0.67/0.61	9.2×10^{-4}	1.29 (1.11-1.49)	0.67/0.61	0.01	1.29 (1.06-1.57)	0.67/0.61	4.2×10^{-3}	1.28 (1.08-1.52)	0.96
rs4977574	G	9	22,088,574	0.49/0.42	3.5×10^{-5}	1.35 (1.17-1.56)	0.48/0.42	5.2×10^{-3}	1.30 (1.08-1.57)	0.50/0.42	4.2×10^{-5}	1.39 (1.18-1.64)	0.65
rs2891168	G	9	22,088,619	0.49/0.42	4.8×10^{-5}	1.35 (1.17-1.56)	0.48/0.42	7.0×10^{-3}	1.29 (1.07-1.56)	0.50/0.42	9.5×10^{-5}	1.38 (1.18-1.63)	0.61
rs1333042	G	9	22,093,813	0.68/0.62	4.4×10^{-4}	1.31 (1.13-1.52)	0.68/0.62	5.3×10^{-3}	1.32 (1.09-1.61)	0.68/0.62	2.7×10^{-3}	1.30 (1.09-1.54)	0.90
rs17761446	T	9	22,108,102	0.81/0.80	0.36	1.09 (0.91-1.30)	0.80/0.80	0.89	1.02 (0.81-1.28)	0.82/0.80	0.22	1.14 (0.93-1.40)	0.48
rs1333048	G	9	22,115,347	0.51/0.45	1.3×10^{-3}	1.26 (1.10-1.46)	0.49/0.45	0.07	1.19 (0.99-1.43)	0.52/0.45	9.7×10^{-4}	1.32 (1.12-1.55)	0.45
rs1333049	C	9	22,115,503	0.50/0.45	1.2×10^{-3}	1.27 (1.10-1.46)	0.49/0.45	0.05	1.20 (1.00-1.45)	0.51/0.45	1.1×10^{-3}	1.31 (1.11-1.54)	0.51
rs10965245	T	9	22,120,515	0.44/0.44	0.96	1.00 (0.87-1.16)	0.45/0.44	0.70	1.04 (0.86-1.25)	0.44/0.44	0.83	0.98 (0.83-1.16)	0.66
rs2891169	A	9	22,121,825	0.40/0.39	0.65	1.03 (0.89-1.20)	0.40/0.39	0.86	1.02 (0.84-1.23)	0.40/0.39	0.60	1.05 (0.89-1.23)	0.83
rs2383208	A	9	22,122,076	0.57/0.56	0.41	1.06 (0.92-1.23)	0.57/0.56	0.55	1.06 (0.88-1.28)	0.57/0.56	0.45	1.07 (0.90-1.26)	0.97
rs10811661	T	9	22,124,094	0.55/0.54	0.43	1.06 (0.92-1.22)	0.55/0.54	0.68	1.04 (0.86-1.25)	0.56/0.54	0.40	1.07 (0.91-1.26)	0.81
rs10757283	A	9	22,124,172	0.67/0.65	0.41	1.07 (0.92-1.24)	0.67/0.65	0.39	1.09 (0.89-1.33)	0.66/0.65	0.58	1.05 (0.88-1.25)	0.79
rs1333051	A	9	22,126,489	0.19/0.18	0.63	1.05 (0.87-1.26)	0.21/0.18	0.16	1.18 (0.94-1.49)	0.18/0.18	0.71	0.96 (0.78-1.19)	0.21

rs7022662	C	9	22,137,715	0.59/0.58	0.56	1.04 (0.90-1.21)	0.59/0.58	0.72	1.04 (0.86-1.25)	0.59/0.58	0.56	1.05 (0.89-1.24)	0.91
rs12341394	C	9	22,138,055	0.72/0.71	0.43	1.07 (0.91-1.25)	0.72/0.71	0.61	1.06 (0.86-1.30)	0.72/0.71	0.45	1.07 (0.90-1.28)	0.92
rs7864275	G	9	22,151,212	0.65/0.64	0.75	1.02 (0.88-1.19)	0.64/0.64	0.96	0.99 (0.82-1.21)	0.65/0.64	0.62	1.04 (0.88-1.24)	0.71
rs10965266	G	9	22,151,494	0.58/0.57	0.45	1.06 (0.91-1.22)	0.58/0.57	0.59	1.05 (0.87-1.27)	0.58/0.57	0.49	1.06 (0.90-1.25)	0.96
rs10965267	T	9	22,151,828	0.96/0.95	0.65	1.08 (0.77-1.53)	0.95/0.95	0.63	0.90 (0.59-1.38)	0.96/0.95	0.29	1.25 (0.83-1.87)	0.29
rs7863846	C	9	22,158,128	0.71/0.70	0.68	1.03 (0.88-1.21)	0.71/0.70	0.61	1.05 (0.86-1.29)	0.70/0.70	0.83	1.02 (0.85-1.22)	0.81
rs828580	A	9	22,158,464	0.96/0.96	0.93	1.02 (0.71-1.45)	0.95/0.96	0.57	0.88 (0.56-1.37)	0.96/0.96	0.56	1.13 (0.74-1.72)	0.42
rs10757292	G	9	22,166,961	0.99/0.99	0.27	1.43 (0.76-2.73)	0.99/0.99	0.30	1.61 (0.66-3.92)	0.99/0.99	0.44	1.34 (0.65-2.77)	0.77
rs10965290	A	9	22,194,445	0.02/0.02	0.18	1.42 (0.86-2.35)	0.03/0.02	0.15	1.57 (0.85-2.90)	0.02/0.02	0.35	1.31 (0.74-2.33)	0.71
rs1679014	A	9	22,197,037	0.04/0.03	0.73	1.07 (0.73-1.58)	0.02/0.03	0.07	0.57 (0.3-1.05)	0.05/0.03	0.10	1.41 (0.94-2.13)	0.01

[a] Risk allele obtained by the results of POAG vs Control.

[b] Risk allele frequency in POAG, HPG or NPG/Control.

[c] *P* value of Cochran's Q heterogeneity test between POAG/HPG vs Control and POAG/NPG vs Control.

[d] These SNPs passed the Bonferroni correction threshold in the both of POAG vs Control and POAG/NPG vs Control.
