# Supplementary material for: Common Variants in CDKN2B-AS1 Associated with Optic-Nerve Vulnerability of Glaucoma Identified by Genome-Wide Association Studies in Japanese
Source: PLoS One. 2012 Mar 12;7(3):e33389. doi: 10.1371/journal.pone.0033389 (PMC3299784; doi:10.1371/journal.pone.0033389)
Supplement: Table S4 — Combined GWAS results in 9p21.3 locus. (PDF) [file pone.0033389.s010.pdf]

Table S4

SNP	POAG vs Control				POAG/HPG vs Control				POAG/NPG vs Control			
	Freq.[a]	*P*[b]	OR (95% CI)	*HetP*[c]	Freq.[a]	*P*[b]	OR (95% CI)	*HetP*[c]	Freq.[a]	*P*[b]	OR (95% CI)	*HetP*[c]
rs4977749	0.55/0.54	0.72	1.02 (0.90-1.17)	0.68	0.55/0.54	0.89	1.01 (0.84-1.23)	0.70	0.55/0.54	0.68	1.03 (0.89-1.20)	0.74
rs2518713	0.55/0.54	0.67	1.03 (0.91-1.17)	0.66	0.55/0.54	0.84	1.02 (0.85-1.22)	0.63	0.55/0.54	0.64	1.04 (0.89-1.20)	0.77
rs7864029	0.55/0.54	0.58	1.04 (0.91-1.18)	0.65	0.55/0.54	0.90	1.01 (0.83-1.23)	0.58	0.55/0.54	0.47	1.06 (0.91-1.22)	0.77
rs717326	0.11/0.08	4.4×10^{-3}	1.36 (1.10-1.69)	0.31	0.10/0.08	0.02	1.37 (1.05-1.78)	0.82	0.11/0.08	0.01	1.37 (1.08-1.75)	0.16
rs3731201	0.02/0.01	0.03	1.91 (1.07-3.39)	0.79	0.02/0.01	0.06	1.92 (0.96-3.85)	0.98	0.02/0.01	0.06	1.87 (0.97-3.62)	0.62
rs643319	0.68/0.62	3.5×10^{-5}	1.30 (1.15-1.48)	0.02	0.66/0.62	0.11	1.14 (0.97-1.34)	0.08	0.71/0.62	9.9×10^{-7}	1.45 (1.25-1.68)	0.07
rs7044859	0.69/0.62	3.7×10^{-5}	1.30 (1.15-1.48)	0.03	0.66/0.62	0.09	1.15 (0.98-1.35)	0.11	0.71/0.62	1.6×10^{-6}	1.44 (1.24-1.67)	0.08
**rs523096**[d]	0.89/0.83	1.6×10^{-10}	1.76 (1.48-2.10)	0.42	0.89/0.83	4.6×10^{-5}	1.59 (1.27-1.99)	0.86	0.90/0.83	1.9×10^{-9}	1.91 (1.55-2.35)	0.31
**rs518394**[d]	0.89/0.83	5.8×10^{-10}	1.74 (1.46-2.07)	0.34	0.89/0.83	9.9×10^{-5}	1.56 (1.25-1.96)	0.75	0.90/0.83	4.5×10^{-9}	1.89 (1.53-2.33)	0.26
rs10757264	0.68/0.62	3.2×10^{-5}	1.31 (1.15-1.48)	0.02	0.66/0.62	0.10	1.15 (0.98-1.34)	0.09	0.71/0.62	1.0×10^{-6}	1.44 (1.25-1.67)	0.08
rs7049105	0.68/0.62	2.0×10^{-4}	1.27 (1.12-1.44)	0.04	0.65/0.62	0.24	1.10 (0.94-1.30)	0.11	0.70/0.62	3.7×10^{-6}	1.42 (1.23-1.65)	0.15
rs10965215	0.69/0.63	3.7×10^{-5}	1.30 (1.15-1.48)	0.03	0.66/0.63	0.11	1.14 (0.97-1.34)	0.11	0.71/0.63	1.0×10^{-6}	1.45 (1.25-1.68)	0.09
**rs564398**[d]	0.90/0.83	1.4×10^{-10}	1.77 (1.49-2.11)	0.47	0.89/0.83	6.8×10^{-5}	1.58 (1.26-1.97)	0.90	0.90/0.83	9.6×10^{-10}	1.94 (1.57-2.40)	0.37
**rs7865618**[d]	0.90/0.83	9.0×10^{-11}	1.78 (1.50-2.12)	0.39	0.89/0.83	5.0×10^{-5}	1.59 (1.27-1.99)	0.85	0.90/0.83	8.1×10^{-10}	1.94 (1.57-2.40)	0.28
rs10965219	0.69/0.62	9.8×10^{-6}	1.33 (1.17-1.51)	0.03	0.66/0.62	0.07	1.16 (0.99-1.36)	0.12	0.71/0.62	2.7×10^{-7}	1.48 (1.27-1.71)	0.10

rs17694572	0.02/0.01	0.02	2.10 (1.13-3.89)	0.91	0.02/0.01	0.05	2.12 (1.01-4.46)	0.96	0.02/0.01	0.04	2.06 (1.02-4.16)	0.81
rs16905599	0.06/0.05	0.05	1.32 (1.00-1.74)	0.16	0.07/0.05	0.02	1.48 (1.06-2.06)	0.12	0.06/0.05	0.28	1.20 (0.86-1.67)	0.54
rs12555547	0.93/0.92	0.18	1.18 (0.93-1.50)	0.40	0.94/0.92	0.18	1.24 (0.91-1.69)	0.59	0.93/0.92	0.36	1.14 (0.86-1.52)	0.42
rs9632884	0.73/0.67	2.5×10^{-5}	1.32 (1.16-1.51)	0.96	0.73/0.67	2.3×10^{-4}	1.37 (1.16-1.62)	0.93	0.72/0.67	1.0×10^{-3}	1.29 (1.11-1.50)	0.93
rs17761197	0.82/0.81	0.63	1.04 (0.88-1.23)	0.20	0.80/0.81	0.55	0.94 (0.76-1.15)	0.18	0.83/0.81	0.19	1.13 (0.94-1.37)	0.55
rs6475606	0.70/0.64	6.7×10^{-5}	1.30 (1.14-1.47)	0.73	0.71/0.64	2.9×10^{-4}	1.35 (1.15-1.59)	0.85	0.69/0.64	2.6×10^{-3}	1.26 (1.08-1.46)	0.78
rs10757272	0.68/0.61	9.4×10^{-6}	1.33 (1.17-1.50)	0.50	0.68/0.61	7.8×10^{-4}	1.31 (1.12-1.54)	0.80	0.68/0.61	8.2×10^{-5}	1.34 (1.16-1.55)	0.37
rs4977574	0.49/0.43	1.6×10^{-5}	1.30 (1.15-1.47)	0.36	0.48/0.43	0.01	1.22 (1.05-1.42)	0.26	0.51/0.43	8.8×10^{-6}	1.37 (1.19-1.58)	0.79
rs2891168	0.49/0.43	2.2×10^{-5}	1.30 (1.15-1.46)	0.38	0.48/0.43	0.01	1.21 (1.04-1.41)	0.29	0.51/0.43	1.0×10^{-5}	1.37 (1.19-1.57)	0.80
rs1333042	0.69/0.62	2.3×10^{-6}	1.36 (1.20-1.54)	0.43	0.68/0.62	3.1×10^{-4}	1.34 (1.14-1.58)	0.80	0.69/0.62	2.7×10^{-5}	1.37 (1.18-1.59)	0.28
rs17761446	0.80/0.80	0.87	1.02 (0.85-1.22)	0.18	0.79/0.80	0.55	0.94 (0.77-1.15)	0.27	0.81/0.80	0.43	1.08 (0.90-1.29)	0.33
rs1333048	0.51/0.45	1.4×10^{-4}	1.26 (1.12-1.42)	0.99	0.50/0.45	0.03	1.19 (1.02-1.38)	0.94	0.52/0.45	6.3×10^{-5}	1.33 (1.16-1.53)	0.84
rs1333049	0.51/0.45	8.3×10^{-5}	1.27 (1.13-1.44)	0.91	0.50/0.45	0.02	1.21 (1.04-1.40)	0.94	0.52/0.45	6.6×10^{-5}	1.33 (1.16-1.53)	0.80
rs10965245	0.43/0.43	0.89	1.01 (0.87-1.17)	0.87	0.44/0.43	0.68	1.04 (0.88-1.22)	0.97	0.43/0.43	0.95	0.99 (0.80-1.23)	0.81
rs2891169	0.41/0.40	0.92	1.01 (0.86-1.18)	0.54	0.40/0.40	0.93	0.99 (0.79-1.24)	0.65	0.41/0.40	0.80	1.02 (0.87-1.20)	0.60
rs2383208	0.58/0.57	0.63	1.03 (0.91-1.17)	0.47	0.58/0.57	0.87	1.02 (0.84-1.22)	0.45	0.58/0.57	0.57	1.04 (0.90-1.21)	0.66
rs10811661	0.56/0.55	0.41	1.05 (0.93-1.19)	0.88	0.56/0.55	0.63	1.04 (0.88-1.23)	0.99	0.56/0.55	0.42	1.06 (0.92-1.23)	0.82
rs10757283	0.66/0.65	0.23	1.08 (0.95-1.23)	0.72	0.67/0.65	0.13	1.13 (0.96-1.34)	0.51	0.66/0.65	0.60	1.04 (0.89-1.22)	0.88
rs1333051	0.19/0.18	0.57	1.05 (0.89-1.24)	0.97	0.20/0.18	0.10	1.18 (0.97-1.43)	0.95	0.17/0.18	0.64	0.90 (0.78-1.16)	0.89

rs7022662	0.59/0.57	0.14	1.10 (0.97-1.24)	0.25	0.58/0.57	0.52	1.05 (0.90-1.24)	0.76	0.60/0.57	0.08	1.14 (0.98-1.31)	0.10
rs12341394	0.72/0.71	0.21	1.09 (0.95-1.25)	0.61	0.72/0.71	0.58	1.05 (0.88-1.26)	0.95	0.73/0.71	0.14	1.13 (0.96-1.32)	0.32
rs7864275	0.65/0.64	0.48	1.05 (0.92-1.19)	0.61	0.63/0.64	0.82	0.98 (0.82-1.17)	0.78	0.66/0.64	0.16	1.11 (0.96-1.29)	0.20
rs10965266	0.58/0.56	0.11	1.10 (0.98-1.25)	0.31	0.57/0.56	0.39	1.07 (0.91-1.25)	0.78	0.59/0.56	0.08	1.14 (0.99-1.31)	0.14
rs10965267	0.95/0.95	0.67	1.07 (0.78-1.48)	0.93	0.94/0.95	0.72	0.93 (0.62-1.40)	0.83	0.96/0.95	0.28	1.22 (0.85-1.74)	0.85
rs7863846	0.71/0.70	0.39	1.06 (0.93-1.22)	0.54	0.71/0.70	0.71	1.03 (0.86-1.24)	0.77	0.72/0.70	0.28	1.09 (0.93-1.28)	0.18
rs828580	0.95/0.96	0.63	0.92 (0.66-1.28)	0.36	0.95/0.96	0.25	0.80 (0.55-1.17)	0.52	0.96/0.96	0.91	1.04 (0.56-1.90)	0.47
rs10757292	0.99/0.99	0.57	1.21 (0.63-2.29)	0.38	0.99/0.99	0.80	1.15 (0.39-3.40)	0.25	0.99/0.99	0.56	1.26 (0.58-2.75)	0.77
rs10965290	0.02/0.02	0.77	1.09 (0.60-2.00)	0.01	0.02/0.02	0.57	1.21 (0.63-2.32)	0.10	0.02/0.02	0.92	0.99 (0.81-1.20)	0.00
rs1679014	0.03/0.04	0.73	0.93 (0.62-1.39)	0.16	0.02/0.04	0.07	0.64 (0.40-1.04)	0.47	0.04/0.04	0.54	1.14 (0.76-1.72)	0.02

[a] Risk allele frequency in combined POAG, HPG or NPG/combined Control.

[b] *P* value of combined 2 GWAS results by Mantel-Haenszel test.

[c] *P* value of Cochran's Q heterogeneity test between two GWAS data set.

[d] These SNPs passed the Bonferroni correction threshold in the both of POAG vs Control and POAG/NPG vs Control.
