# Supplementary material for: Common Variants in CDKN2B-AS1 Associated with Optic-Nerve Vulnerability of Glaucoma Identified by Genome-Wide Association Studies in Japanese
Source: PLoS One. 2012 Mar 12;7(3):e33389. doi: 10.1371/journal.pone.0033389 (PMC3299784; doi:10.1371/journal.pone.0033389)
Supplement: Table S5 — Association results of Present GWAS for previously reported SNPs. (PDF) [file pone.0033389.s011.pdf]

Table S5

Ref.[a]	Ethnicity[b]	SNP :risk allele	Chr.	Postion	Nearest gene	POAG vs Control			POAG/HPG vs Control			POAG/NPG vs Control		
						Freq.[c]	*P*	OR (95% CI)	Freq.[c]	*P*	OR (95% CI)	Freq.[c]	*P*	OR (95% CI)
A	Asian (Japanese)	rs547984 :A	1	236,163,509	*ZP4*	0.507 / 0.473	0.07	1.14 (0.99-1.32)	0.502 / 0.473	0.23	1.12 (0.93-1.35)	0.510 / 0.473	0.08	1.16 (0.99-1.36)
		rs540782 :C	1	236,163,662	*ZP4*	0.507 / 0.475	0.08	1.14 (0.99-1.31)	0.502 / 0.475	0.27	1.11 (0.92-1.34)	0.511 / 0.475	0.08	1.15 (0.98-1.36)
		rs693421 :A	1	236,165,713	*ZP4*	0.503 / 0.463	0.03	1.18 (1.02-1.36)	0.495 / 0.463	0.17	1.14 (0.95-1.37)	0.508 / 0.463	0.03	1.20 (1.02-1.41)
		rs2499601 :C	1	236,171,518	*ZP4*	0.500 / 0.463	0.04	1.16 (1.01-1.34)	0.495 / 0.463	0.13	1.15 (0.96-1.39)	0.503 / 0.463	0.03	1.20 (1.02-1.41)
		rs7081455 :A	10	20,678,891	*PLXDC2*	0.803 / 0.788	0.31	1.10 (0.92-1.31)	0.811 / 0.788	0.23	1.15 (0.91-1.46)	0.798 / 0.788	0.56	1.06 (0.87-1.30)
		rs7961953 :C	12	81,615,967	*TMTC2*	0.314 / 0.287	0.11	1.14 (0.97-1.33)	0.276 / 0.287	0.59	1.06 (0.86-1.30)	0.340 / 0.287	6.3×10^{-3}	1.28 (1.07-1.52)
B	Asian (Japanese)	rs3213787 :C	2	45,500,328	*SRBD1*	0.882 / 0.876	0.59	1.06 (0.85-1.32)	0.889 / 0.876	0.38	1.14 (0.85-1.52)	0.878 / 0.876	0.89	1.02 (0.79-1.30)
		rs735860 :C	6	53,231,077	*ELOVL5*	0.451 / 0.423	0.11	1.12 (0.97-1.30)	0.458 / 0.423	0.14	1.15 (0.96-1.39)	0.447 / 0.423	0.23	1.11 (0.94-1.30)
C	Caucasian (Icelandian)	rs4236601 :A	7	115,949,965	*CAV1*	N / A[d]								
D	Caucasian (Australian)	rs4656461 :G	1	163,953,829	*TMCO1*	N / A[d]								
		rs7518099 :C	1	164,003,504	*TMCO1*	0.004 / 0.002	0.33	1.93 (0.54-6.87)	0.005 / 0.002	0.36	2.09 (0.47-9.21)	0.004 / 0.002	0.43	1.82 (0.45-7.39)
		rs10120688 :A	9	22,046,499	*CDKN2BAS*	0.696 / 0.613	1.6×10^{-6}	1.44 (1.24-1.68)	0.667 / 0.613	0.02	1.27 (1.04-1.54)	0.715 / 0.613	2.5×10^{-7}	1.58 (1.33-1.88)
		rs4977756 :A	9	22,058,652	*CDKN2BAS*	N / A[d]								

[a] References: A - Nakano et al. Proc Natl Acad Sci U S A 106: 12838-12842 (2009).
B - Meguro et al. Ophthalmology 117: 1331-1338 e1335 (2010).
C - Thorleifsson et al. Nat Genet 42: 906-909 (2010).
D - Burdon et al. Nat Genet 43: 574-578 (2011).

[b] Ethnicities of the populations used in each first or discovery GWAS stage.

[c] Risk allele frequency in POAG, HPG, or NPG/Control.

[d] SNPs not designed into the Affymetrix SNP 6.0 Chip.
