# Supplementary material for: Common Variants in CDKN2B-AS1 Associated with Optic-Nerve Vulnerability of Glaucoma Identified by Genome-Wide Association Studies in Japanese
Source: PLoS One. 2012 Mar 12;7(3):e33389. doi: 10.1371/journal.pone.0033389 (PMC3299784; doi:10.1371/journal.pone.0033389)
Supplement: Table S6 — Characteristics of the samples. (PDF) [file pone.0033389.s012.pdf]

Table S6

			n	Age	P [a]	female/male	P [b]
Present GWAS							
	POAG		833	61.5 ± 13.7	8.7×10^{-5}	1.1	$< 1.0 \times 10^{-6}$
		HPG	330	63.0 ± 12.9	2.0×10^{-6}	0.9	$< 1.0 \times 10^{-6}$
		NPG	503	60.6 ± 14.1	0.03	1.3	3.2×10^{-4}
	Control		686	58.8 ± 13.5		2.0	
Previous GWAS							
	POAG		411	64.7 ± 13.3	$< 1.0 \times 10^{-6}$	1.0	0.23
		HPG	215	65.7 ± 12.4	$< 1.0 \times 10^{-6}$	0.8	0.02
		NPG	196	63.6 ± 14.1	$< 1.0 \times 10^{-6}$	1.3	0.83
	Control		289	51.2 ± 13.9		1.3	

[a] *P* value of Student's t test for case and control comparisons.

[b] *P* value of χ^2 test for case and control comparisons.
