# Supplementary material for: Common Variants in CDKN2B-AS1 Associated with Optic-Nerve Vulnerability of Glaucoma Identified by Genome-Wide Association Studies in Japanese
Source: PLoS One. 2012 Mar 12;7(3):e33389. doi: 10.1371/journal.pone.0033389 (PMC3299784; doi:10.1371/journal.pone.0033389)
Supplement: Supporting Information S1 — Quality control for genotyping. (PDF) [file pone.0033389.s013.pdf]

Supporting Information S1

Quality control for genotyping

We summarized the procedure of the quality control (QC) as a flow chart shown below (Figure A).

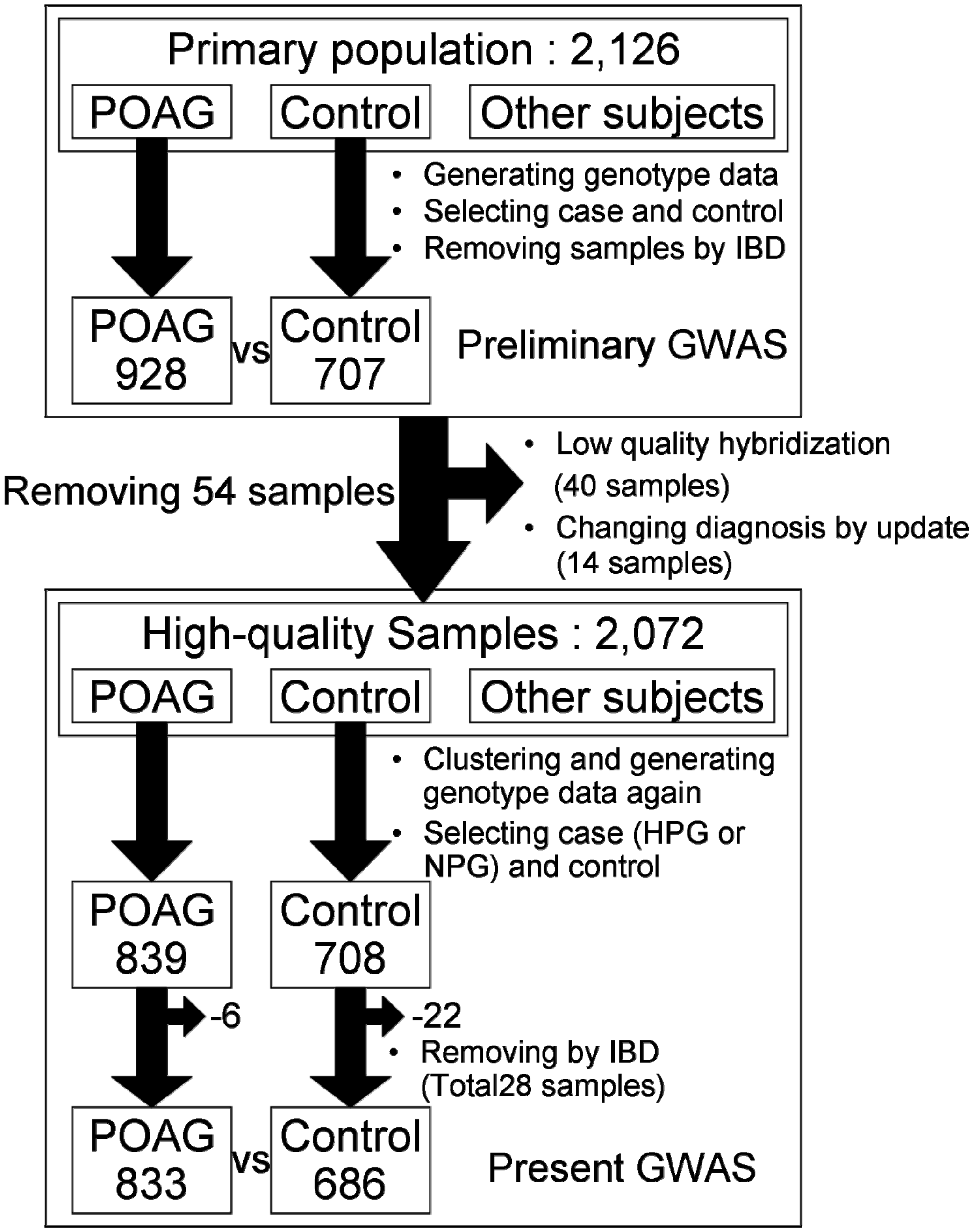

Figure A. Flow chart of the quality control procedure

In order to obtain the data for the present GWAS, we first genotyped for 906,600 SNPs of 2,126 Japanese subjects (referred to as "primary population"), including the subjects for different studies, by Genome-Wide Human SNP Array 6.0 (Affymetrix, Santa Clara, CA) according to the manufacturer's instructions. Based on the QC process of the system, Contrast QC and QC Call Rate calculated by QC algorithm were 2.23 ± 0.38 and 96.72 ± 1.92, respectively. Then, genotype calls were generated by using the Birdseed v2 algorithm (Affymetrix), and the preliminary GWAS was performed by selecting case and control samples from the primary population.

However, we observed two unreasonable results as below:

1) In our preliminary analysis of identity-by-descent (IBD) using the primary population to exclude genetically related subjects, we observed thousands of family combinations suggesting weak, but unignorable correlations from a part of the samples to the vast majority of the rest of the samples (data not shown).
2) In our preliminary GWAS using 928 POAG and 707 controls, which were selected from 2,126 subjects after removing the related subjects by IBD analysis, we obtained no fewer than 59 unreasonable SNPs that passed the Bonferroni correction threshold (Figure B).

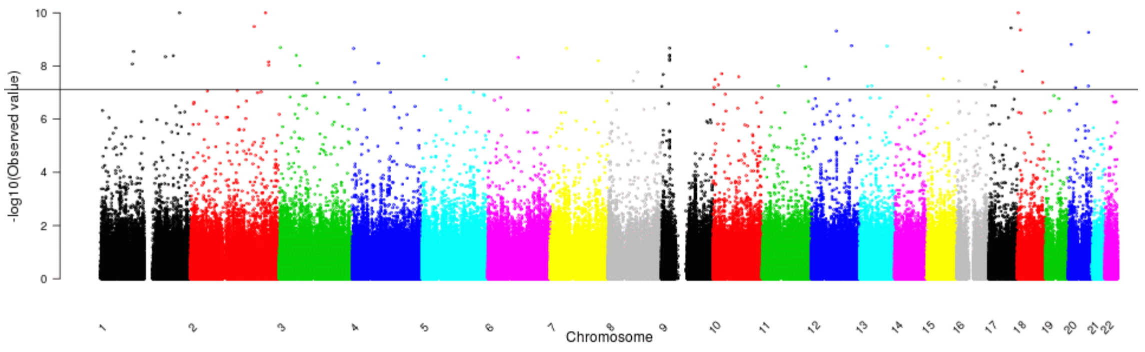

Figure B. **Manhattan plot of the preliminary GWAS**

Above two observations were considered to be false-positive signals, mainly due to the inclusion of poorly clustered genotyping data as shown in Figure C. In addition, most of these SNPs with low *p*-value seemed to be false-positive because they appeared

as a single signal, but not as a cluster, and the surrounding SNPs were not so significant.

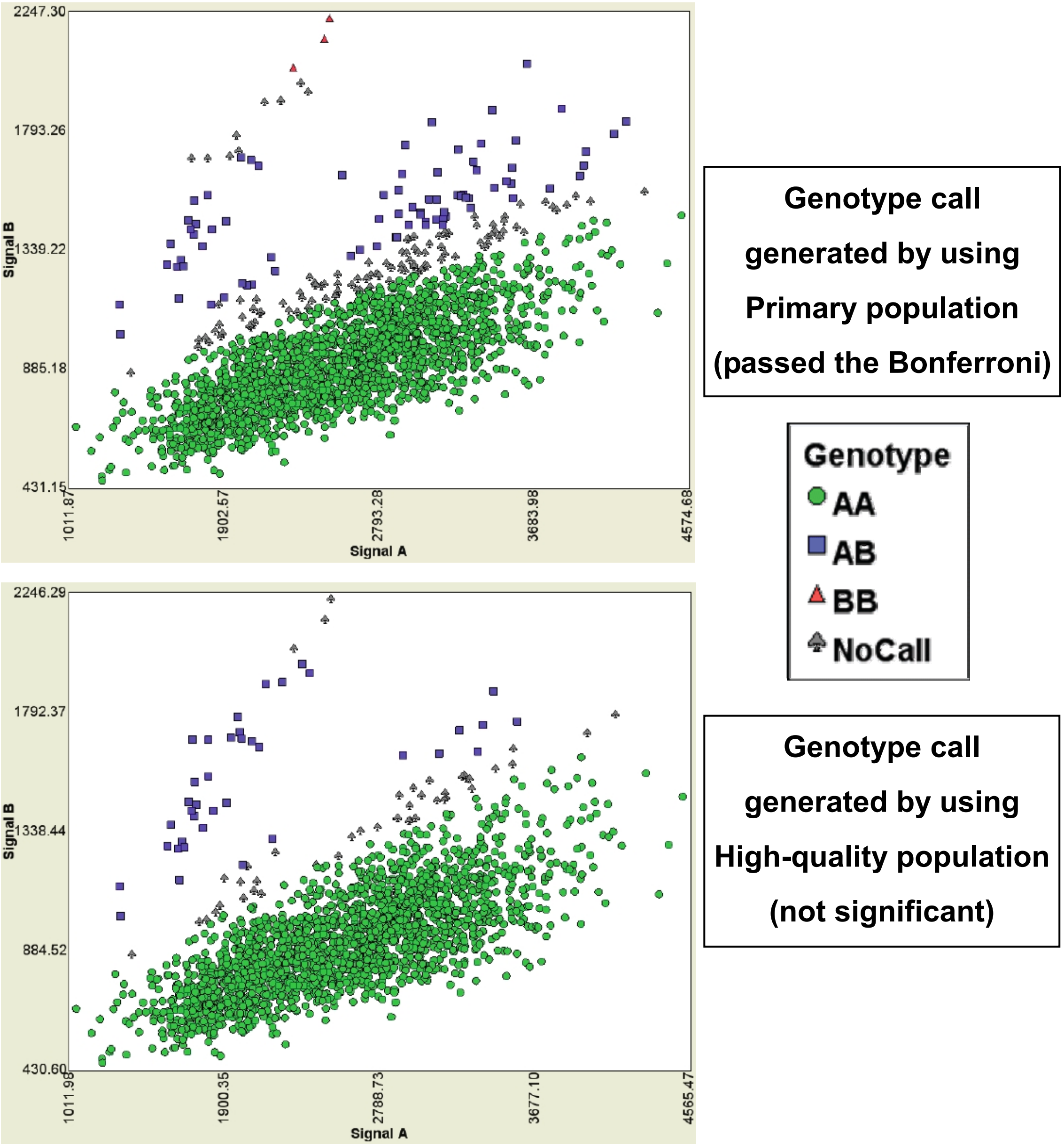

Figure C. Example of 2D-cluster plots generated for the same SNP

Therefore, we decided to apply more stringent filters than the filter recommended by Affymetrix (Figure D). As a result, 40 subjects were removed based on our original QC filters, instead of using the less-stringent Affymetrix default filter (Contrast QC ≥0.4), as follows: (i) QC Call Rate ≥90% and (ii) Contrast QC ≥1.0 (Figure D).

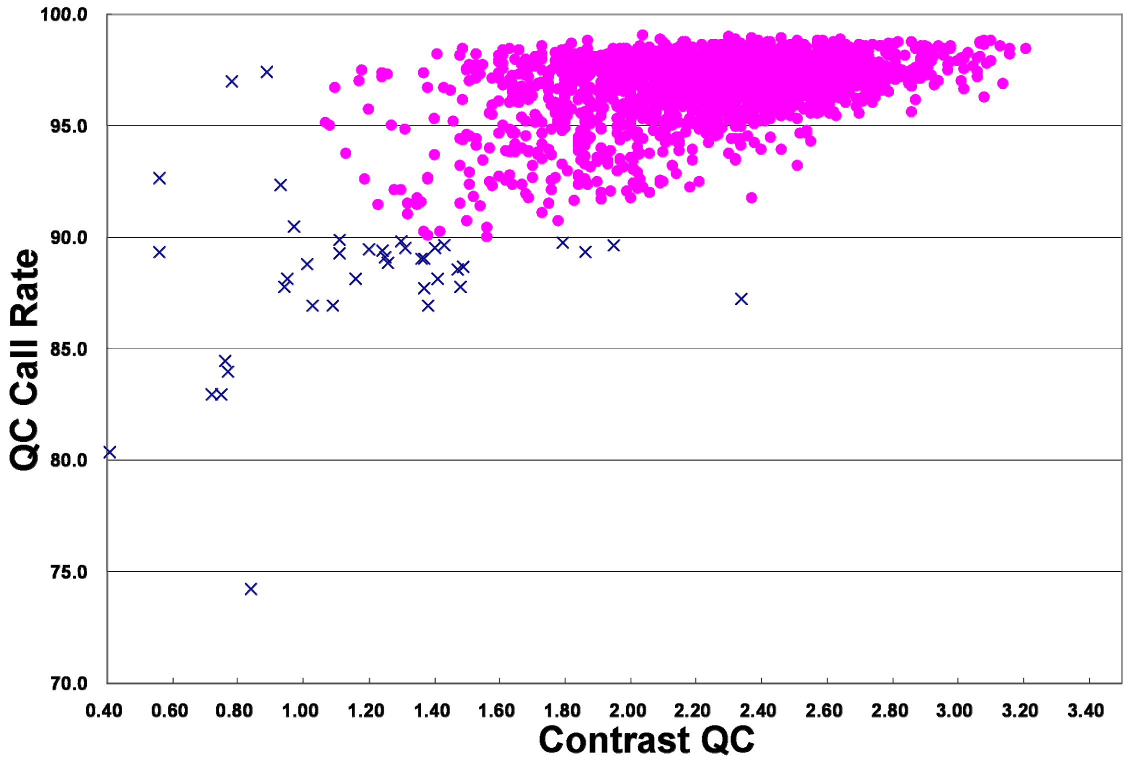

Figure D. Scatter plot for quality control of the present GWAS data set. Each plot indicates the qualities (QC Call Rate and Contrast QC) of array hybridization for each sample. Although all of the plots satisfied the quality of manufacturer's recommendation, we decided to select higher-quality samples (shown in pink dots) by applying our original stringent filters (QC Call Rate ≥90% and Contrast QC ≥1.0). Therefore, low-quality samples (shown in blue crosses) were excluded from the calculation for obtaining genotype data.

Moreover, we paid strict attention to the updated clinical record of each subject. As a result, 14 subjects were removed because of the changes of diagnosis in a follow-up survey after the array experiments. Finally, after removing 54 above subjects from the primary population, the remaining 2,072 high-quality subjects (Contrast QC 2.25 ± 0.35 and QC Call Rate 96.88 ± 1.44) were applied to generate genotype call data again by using the Birdseed v2 algorithm. In addition, a part of the POAG subjects were also excluded from the case-control study because of the ambiguous diagnosis of classifying into NPG or HPG categories.
